# Supplementary material for: Correcting for volunteer bias in GWAS increases SNP effect sizes and heritability estimates
Source: Nat Commun. 2025 Apr 15;16:3578. doi: 10.1038/s41467-025-58684-8 (PMC12000612; doi:10.1038/s41467-025-58684-8)
Supplement: Supplementary file 1 — Supplementary Information [file 41467_2025_58684_MOESM1_ESM.pdf]

Supplementary notes and figures to: Correcting for volunteer bias in GWAS increases SNP effect sizes and heritability estimates

Contents

S1 Impact of volunteer bias on SNP associations estimated through GWAS 2

S2 Coding of phenotypes 8

S3 GWAS on the inverse probability weights 11

S4 Follow-up of new loci found by WGWAS 12

S5 Robustness analysis of new genome-wide significant loci found in WGWAS 14

S6 WGWAS reduces autosomal heritability of sex 16

S7 Supplementary Figures 17

S1 Impact of volunteer bias on SNP associations estimated through GWAS

Non-random sample selection may bias single nucleotide polymorphism (SNP) associations in various directions, depending on the associations between the outcome Y, the SNP, and their associations with selection into the data set. Here, we provide some simulations to illustrate how GWAS associations and their resulting SNP-based heritabilities are biased under various scenarios of selection into the data set.

S1.1 Setup

First, we consider how selection biases estimates when studying a continuous phenotype, Y. To this end, we simulate a phenotype on our UKB data using LDAK software, using only the 10th chromosome to reduce the computational burden of these simulations.[1] We use a SNP-based heritability of 20%. The number of causal SNPs for the trait that we consider varies between 1 (monogenic), 10, 200, 2000, and all SNPs (omnigenic).

From the UKB sample, we simulate a hypothetical sample (S) with a participation rate of 5% (i.e. $N = 19,124$), assuming various scenarios of selection as described below. To ensure that none of our simulation results are driven by sampling error, we report average results based on 30 iterations of these simulations.

Without loss of generality, we assume that Y positively influences the probability of being selected into the sample. We consider the following scenarios to resemble how selection into the UKB could be based on the phenotype and/or the genotype:

Scenario 1: Phenotype-related selection – Under this scenario, those with higher values of the phenotype Y (e.g., higher educated people) are more likely to volunteer into the sample. This results in non-representativeness of the genotype in the selected sample, but only because of moderation through the phenotype Y. To illustrate this in our simulations, we model selection into the sample S as follows:

$$S = \beta Y + \varepsilon \tag{4}$$

with ε normally distributed with mean zero and variance one. Next, we define our sample as the 5% of this population with the largest simulated values S. We pick various values for β: $\beta \in \{0, 0.2, 0.4, 0.6, 0.8, 1\}$. The higher the value of β, the higher the discrepancy between Y in the sample S and the underlying population. When $\beta = 0$, our 5% subsample is randomly selected from the underlying UKB data.

Scenario 2: Phenotype-genotype-related selection – Another scenario, *phenotype-genotype-related selection*, is arguably more concerning. Under this scenario, sample selection is both driven by the phenotype and *independently* by the genotype as well. For example, consider a research design in which a SNP's association with educational attainment is tested. It could be that this SNP influences another phenotype, say, a disease. This disease may prevent people from volunteering in the study whereas education may encourage people to volunteer. In this scenario, collider bias occurs: a correlation between the SNP and education appears even if the SNP does not influence education. The sign of the bias is then hard to predict, as it depends on the sign by which both the phenotype and the SNP of interest influence sample selection.

In reality, it is unlikely that SNPs relating to a phenotype Y would influence participation directly. However, it is plausible that they independently influence a second phenotype, which in turn influences participation. We model this idea by positing that the SNPs that influence Y could influence S independently by modeling an association between a *polygenic index* based on the SNPs that affect Y and our participation propensity S, as follows:

$$PGI_Y = \sum^{J} \gamma_j SNP_j \tag{5}$$

where γ_j is the true causal effect of the jth SNP on Y as given by our simulation. Note that in the monogenic case, the polygenic index reduces to $\gamma_j \cdot SNP_j$. We standardize the

polygenic index to have mean zero and standard deviation one in the UKB data.

Selection is next modeled as

$$S = \beta_1 Y + \beta_2 PGI_Y + \varepsilon \qquad (6)$$

For simplicity, we set $\beta_1 = \beta_2$ (scenario 2a) or $\beta_1 = -\beta_2$ (scenario 2b). Hence, in scenario 2b, Y positively influences participation in the sample, whereas the polygenic index negatively influences participation. Again, those with the highest 5% values of S are included in the sample.

S1.2 Results of simulations

We use our simulated selected samples to assess how selection bias influences GWAS-based results. First, we show how selection affects the association between the phenotype Y and the SNP that is the *top predictor* for this phenotype (as based on the draws of true causal effects). Second, for the cases where Y is a polygenetic trait ($\geq$ 10 causal SNPs), we estimate the SNP-based heritability given by a GWAS of Y within the selected sample, as estimated through LD-score regression.

S1.2.1 Effects on top SNPs

Figure S21 shows that increases in the selection parameter β lead to bias in the estimated associations between the phenotype Y and the top SNP relative to the true association (the black dotted line).

We begin with the simple case where Y is a monogenic trait (1 causal SNP) in Figure S21a. When β is zero, there is no non-random selection, and the estimated association in the sample is equal to the true value under all selection scenarios. When β increases, the difference in the association estimated differs further and further from the true association. Under scenario 1, selection is only influenced by the phenotype. This effectively narrows the distribution

of Y in the sample, pushing the association towards the null, i.e. attenuation bias, and, hence, smaller estimated SNP effect sizes[2]. This could potentially result in false negatives. Under scenario 2a, the bias is also negative (and more severe), again pushing the association between the SNP and the outcome towards the null. Under scenario 2b, the sign of the bias flips and becomes positive, such that the association is overestimated. Hence, this scenario could potentially result in false positives. As under Phenotype-genotype-related selection the bias takes the form of collider bias, the bias can even result in false positives when the true SNP effect size is zero, or can result in incorrect effect sizes (possibly of the opposite sign) for SNPs that *do* affect Y.

When the phenotype is polygenic (Figure S21b-Figure S21e), effects on the top SNP are similar: with attenuation bias under scenario 1, downward bias under scenario 2a, and upward bias under scenario 2b. However, the effect of selection bias on the association of the top SNPs becomes smaller, the more SNPs influence the phenotype. This is intuitive: when more SNPs influence the phenotype, the overall contribution of the top SNP to the phenotype is relatively smaller, such that the effect of selection bias on the association between the top SNP and this phenotype is also smaller in an absolute sense.

S1.2.2 Effects on SNP-based heritabilities

Next, we illustrate how results of the full GWAS are biased by selection: we estimate a GWAS for the simulated phenotype Y in our selected sample S, and estimate its SNP-based heritability through LD-score regression. The resulting heritability estimates are shown in Figure S22 at different values of β and for different numbers of causal SNPs underlying the trait. Here, it becomes clear that, under scenario 1 and 2a, the attenuation bias already discussed in the subsection above translates into *lower* SNP-based heritabilities. However, under scenario 2b, SNP-based heritabilities may be overestimated, similar to the monogenic case illustrated in Figure S21a. This pattern is the most pronounced when the phenotype is highly polygenic (i.e. the case of 2000 causal SNPs or all SNPs being causal, Figure S22b

and Figure S22d). LD-score regression based heritabilities are the most reliable in the cases of 2000 causal SNPs or all causal SNPs, due to the assumption of an underlying polygenic model[22].

S1.3 Binary phenotypes

When studying a binary phenotype, the direction of volunteer bias becomes more difficult to predict. To test this, we again simulated two phenotypes on our UKB data using LDAK for various numbers of causal SNPs on the tenth chromosome, but now converted these phenotypes to binary. The first considered phenotype has prevalence set at 5% and the second at 95%. We next considered how selection on such binary phenotypes influences estimated associations for 1) the top SNP and 2) the SNP-based heritability as estimated through LD-score regression, according to selection scenarios 1, 2a, and 2b, as defined in subsection S1.1. Here, we only show the results for simulations where the phenotype is monogenic, or is influenced by 2000 causal SNPs, as results for other types of genetic architecture were very similar.

Figure S23 shows the effect of selection on the association between the monogenic SNP and a binary phenotype with prevalence 5% (Figure S23a) and 95% (Figure S23b). In the case of *phenotype-based selection (Scenario 1)* It can be seen that when the population prevalence of the phenotype is low (5%), the association between the top SNP and binary outcome is *overestimated*. By contrast, when the population prevalence of the phenotype is high (95%), phenotype-based selection results in an *underestimation* of the SNP effect. When genotype-based selection is also present (Scenario 2a and 2b), bias is pushed down relative to the case of only phenotype-based selection. Similar findings were obtained when 2000 SNPs influence the outcome and the association between the top SNP and the binary phenotype is estimated (Figure S23c, Figure S23d).

Last, these patterns translate to SNP-based heritabilities, with larger SNP-based heritabilities when phenotype prevalence is 5% under phenotype-based selection and low values

of phenotype-genotype-based selection, and lower SNP-based heritabilities when prevalence is 95%, as compared to a model of no selection ($\beta = 0$) (Figure S24).

S2 Coding of phenotypes

S2.1 Age at first birth

Age at first birth was assessed for females only and was derived from data field 2754 ("How old were you when you had your FIRST child?"). Respondents could indicate a numerical value, or could answer "Do not remember" or "Prefer not to answer", in which cases the variable was coded as missing.

S2.2 BMI

We used measured BMI as reported in data field 210001.

S2.3 Breast Cancer

Diabetes was derived from data field 40006 (Type of cancer: ICD10). Cases of breast cancer were defined by codes C50.0-C50.9. As there were very few male cases of breast cancer, we studied this phenotype only for those whose genetic sex was female.

S2.4 Type 1 Diabetes (T1D)

Diabetes was derived from data field 41202 (Diagnoses - main ICD10), and, 41204 (Diagnoses - secondary ICD10). Cases of T1D were defined by codes E10.0-E10.9.

S2.5 Drinks per week

Drinks per week was constructed from data field 1568 (average weekly red wine intake), 1578 (average weekly champagne plus white wine intake), 1588 (average weekly beer plus cider intake), 1598 (average weekly spirits intake), 1608 (average weekly fortified wine intake), and 5364 (average weekly intake of other alcoholic drinks). These values were self-reported. On each question, respondents could indicate "Do not know" or "Prefer not to answer".

We coded values for respondents who filled out these options on any of these questions as missing, with the exception of data field 5346, for which we put a value of zero. Drinks per week was then defined as the sum of all these data fields as reported during the first non-missing wave.

S2.6 Height

We use measured standing height (in cm) as reported in data field 50.

S2.7 Health (self-reported)

For self-reported health, we use data field 2178 ("In general how would you rate your overall health?"). Respondents could answer on a likert scale of 1-4 (1: Excellent, 2: Good, 3: Fair, 4: Poor). We inverted this likert scale such that a higher value implies better self-reported health. Respondents could also indicate "Do not know" or "Prefer not to answer". These instances were coded as missing.

S2.8 Physical Activity

We measure physical activity as a weighted sum of duration of moderate physical activity (data field 894) and vigorous physical activity (data field 914). Both frequencies were self-reported and measured as *minutes per day*, we converted this to minutes per week by multiplying by 7. Next, we converted this measure to the metabolic equivalent of moderate and vigorous activity combined, by multiplying moderate activity by 4, vigorous activity by 8, and taking the sum[4].

S2.9 Severe Obesity

Severe obesity was derived from data field 41202 (Diagnoses - main ICD10), and, 41204 (Diagnoses - secondary ICD10). Cases of severe obesity were defined by codes E66.0-E66.9.

S2.10 Years of Education

For years of education, we follow the coding procedure as in the most recent GWAS for educational attainment[5].

S3 GWAS on the inverse probability weights

In Supplementary Data 4, we list the 7 genomewide significant SNPs found in our GWAS on the IP weights, as well as suggestive top hits ($P \leq 5 \cdot 10^{-5}$, 408 approximately independent SNPs in total). Researchers who study these loci in the UKB, or who find that these loci pop up in hypothesis-free approaches (e.g., GWAS) are advised to use an IP weighting procedure to investigate whether their results are driven by volunteer bias.

Analyzing the results of our GWAS on the IP weights, the quantile-quantile plot of the p-values shows an early lift-off (Figure S3, $\lambda = 1.55$), which implies that volunteer bias is highly polygenic and may potentially impact associations of genetic markers across the genome.

We investigated the 7 top hits for UKB participation in further detail. Moving beyond HapMap3 SNPs, we re-estimated the GWAS on the IP weights for all SNPs found in the UKB that were in linkage disequilibirum ($R^2 > 0.1$ and within a 500 kb window size) with these top hits. Supplementary Figure S14 maps these areas of the genome. We obtained data on SNP-trait associations from the GWAS catalog, which has collected over 400,000 SNP-trait associations at the moment of writing[6]. We only include genomewide significant findings from the catalog ($P < 5 \cdot 10^{-8}$). In Supplementary Figure S14, SNPs that were associated with any other trait as reported in the GWAS catalog are annotated as such. For example, Supplementary Figure S14a shows that SNPs in strong LD with lead SNP rs4399146 on chromosome 1 (one of the seven that significantly associates with our IP weights), have been reported to associate with high-density lipoprotein cholesterol, total blood protein, platelet count, and red blood cell distribution width. For the other 6 SNPs, we find that they tag loci that have been reported to relate to educational attainment, alcohol consumption, hypothyroidism, leukocyte count, lymphocyte count, autoimmune disease, and intelligence.

S4 Follow-up of new loci found by WGWAS

Using WGWAS, we found 3 independent loci that are genome-wide significant for T1D (lead SNPs rs9861858, rs12522568, rs17186868), with associations that differed significantly ($P_H < 5 \cdot 10^{-8}$) from their GWAS counterparts. For breast cancer, we found 1 such new independent genome-wide significant locus (lead SNP rs2306412).

S4.1 Follow-up in dbSNP

First, we used the dbSNP database to understand the regions in which these SNPs were located[7]. Three of these SNPs are intronic: rs12522568 is located on LARP1, rs17186868 is located on CABLES1, and rs2306412 is located on ANXA5. LARP1 plays a central role in immunological processes[42]. Hence, our newly identified association between LARP1 and the autoimmune disease T1D could be potentially interesting for follow-up analyses. The ANXA5 gene plays a role in cancer-related processes such as cellular signal transduction, inflammation, growth and differentiation[43], which may underly the newly found association between this gene and breast cancer.

S4.2 Follow-up in GWAS catalog

To assess whether these loci were tagged in other GWASs, we proceeded as follows. We obtained data on SNP-trait associations from the GWAS catalog, which has collected over 400,000 SNP-trait associations at the moment of this writing.[6] We only include findings from the catalog that were suggestive of significance ($P < 5 \cdot 10^{-5}$).

To assess whether the novel loci we uncovered in WGWAS were reported as genome-wide significant elsewhere, we considered our lead SNP (given by the lowest p-value in the region) and re-estimated the WGWAS on the trait for *all* SNPs that were in linkage disequilibrium with this lead SNP, and were available in the UKB (not just HapMap3 SNPs). Figure S15 and Figure S16 show zoomed in Manhattan plots around these lead SNPs for

type 1 diabetes and breast cancer, respectively. We annotated each SNP with the traits for which significant associations were reported in the GWAS catalog, if any. As can be seen, none of the new SNPs we found tag loci that were previously reported for type 1 diabetes or breast cancer respectively. Thus, the SNPs we identified are novel. rs17186868, found to associate with type 1 diabetes in WGWAS, is in strong linkage disequilibrium $R^2 > 0.9$ with a lead SNP that is associated with BMI-adjusted waist circumference, and in weaker linkage disequilibrium with lead SNPs associated with body height and BMI-adjusted hip circumference. rs12522568, found to associate with type 1 diabetes in WGWAS, shows some evidence of being in linkage disequilibrium with a lead SNP for adolescent idiopathic scoliosis.

S4.3 Follow-up in previously published GWAS

Last, we use one previously published GWAS on T1D[8] and one on Breast Cancer[9] to obtain previously published effect sizes for these SNPs. Both these GWASs were the most recently published meta-analyses of these traits that we could find that had 1) GWAS summary statistics publicly available and 2) did *not* include any UKB data. The GWAS on breast cancer was estimated in 122,977 cases and 105,974 controls of European ancestry. The GWAS on T1D was smaller, with 9,358 cases and 15,705 controls of European ancestry. However, none of the SNPs that we found to be newly genomewide significant in WGWAS show any (marginally) associated effect sizes with their respective phenotype: the p-values were larger than 0.4 in all cases in these previously published GWASs.

S5 Robustness analysis of new genome-wide significant loci found in WGWAS

In this Supplementary Note, we study the degree to which the weighted GWAS results for the loci that we observed to be genome-wide significantly associated with T1D (3 in total) or breast cancer (1 in total) in WGWAS are robust to changes in various modeling assumptions. First, we test whether the differences between weighted and unweighted GWAS analyses also replicate when our control variables are dropped from the regression, or when we change the specification from a linear probability model to a logistic regression. These results are reported in Supplementary Data 7 and 8. These different specifications all yielded similar results, confirming the robustness of the new loci we identified through WGWAS to various modelling assumptions.

Next, we assessed whether the weighted association also replicated with different sets of weights. Our UK Census weights are based on a total of 4,820 predictors. If the weighted associations of the 4 novel SNPs only appear for our precise set of weights, it would cast doubt on the validity of these WGWAS estimates. To test this, we re-estimated different sets of IP weights from UK Census and UKB data (using the pipeline described in ref.[2]). These weights were estimated on different sets of variables underlying their construction: we first based them on a single variable to predict UKB participation, next on interactions between two possible variables, etc. The goal was to find the sparsest specification of variables that could still replicate the weighted associations for each of these SNPs.

For the association between rs2306412 and breast cancer, we show re-estimated versions of the weighted association, based on different sets of IP weights, in Figure S17. To the far left is the unweighted estimate (in red, including a 95% confidence interval). The next coefficient (in blue) displays our effect found in WGWAS, where all predictors are included in weight construction. In green, we show various re-estimates of weighted effect sizes based on weights that include a sparser set of variables. We find that weights based on a single

variable mostly do *not* result in a different estimate compared to an unweighted estimate. However, weights based strictly on the variables of education or region of residence result in an estimate trending away from the null in the direction of our fully weighted estimate: these variables both seem to be important predictors for volunteer bias for this SNP. The last green bar shows that estimating weights on education and region, including all possible two-way interactions between these variables, results in a point estimate that is similar to the genome-wide significant point estimate obtained using our original IP weights. We thus conclude that the type of selection that causes volunteer bias in the association between this locus and breast cancer can mostly be captured by region of residence and educational attainment.

Similarly, when re-estimating the associations between the three newly found loci and diabetes (Figure S18-S20), no weights based on a single variable result in association estimates that lie far away from the unweighted estimate. We therefore next considered weights based on models that include multiple variables (and all their two-way interactions). We conclude that, at all three loci, a model that interacts the three variables of employment status, educational attainment, and self-reported health, is a reasonable specification to replicate the weight estimate of weights that use all variables.

In sum, the new signal at these loci obtained through WGWAS does *not* seem to be driven by any specific modeling assumptions, and also replicates with different (sparser) models underlying IP weight estimation. Both insights point towards the robustness of this signal as identified through WGWAS.

S6 WGWAS reduces autosomal heritability of sex

In the UKB and other volunteer-based data sets, sex is significantly heritable on the autosome, an artifact that is indicative of sex-differential volunteer bias[6]. We compared heritability estimates (on the observed scale), based on WGWAS and GWAS. The GWAS heritability was 0.0113 ($s.e. = 0.0015$, $p < 1 \times 10^{-8}$. For WGWAS, the heritability decreased to 0.0095 ($s.e. = 0.003$, $p = 0.0015$).

Although heritability was reduced after taking volunteer bias into account through WGWAS, significant heritability remains. This suggests that, although the weights do capture volunteer bias present in unweighted genetic associations, some volunteer bias remains.

S7 Supplementary Figures

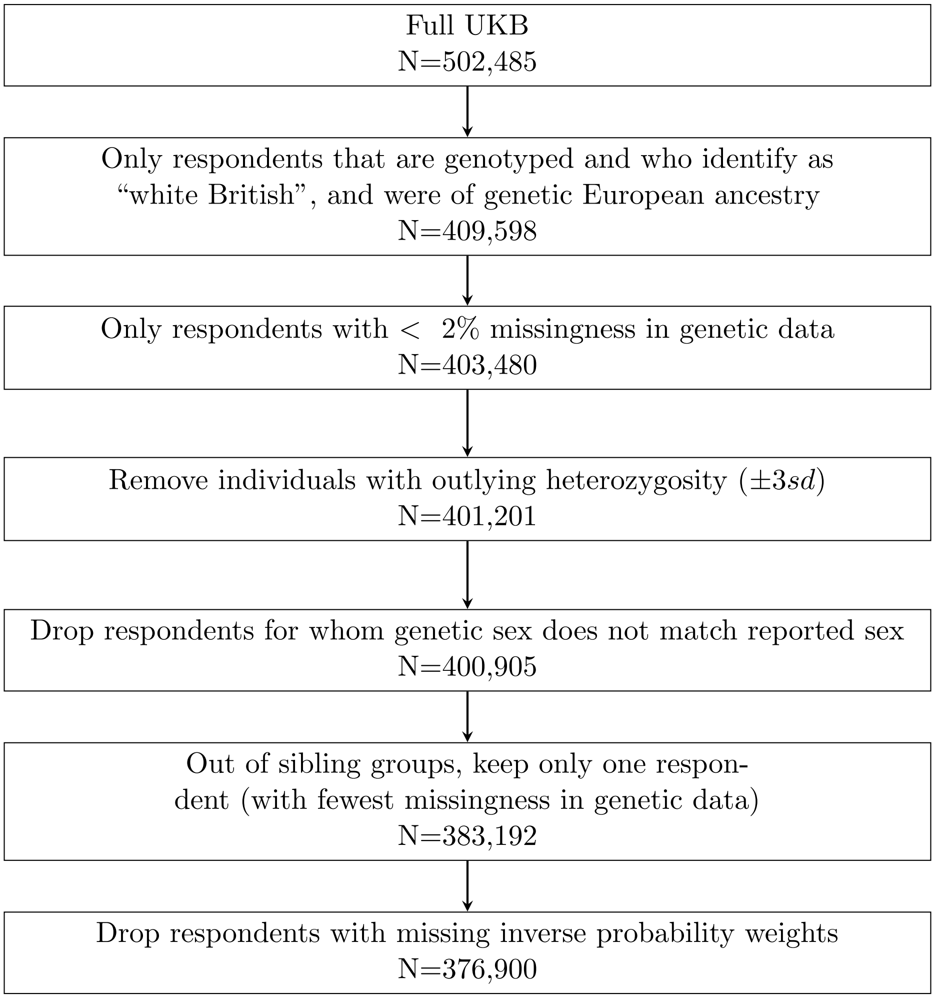

Figure S1: **Summary of sample restrictions made to the UKB**

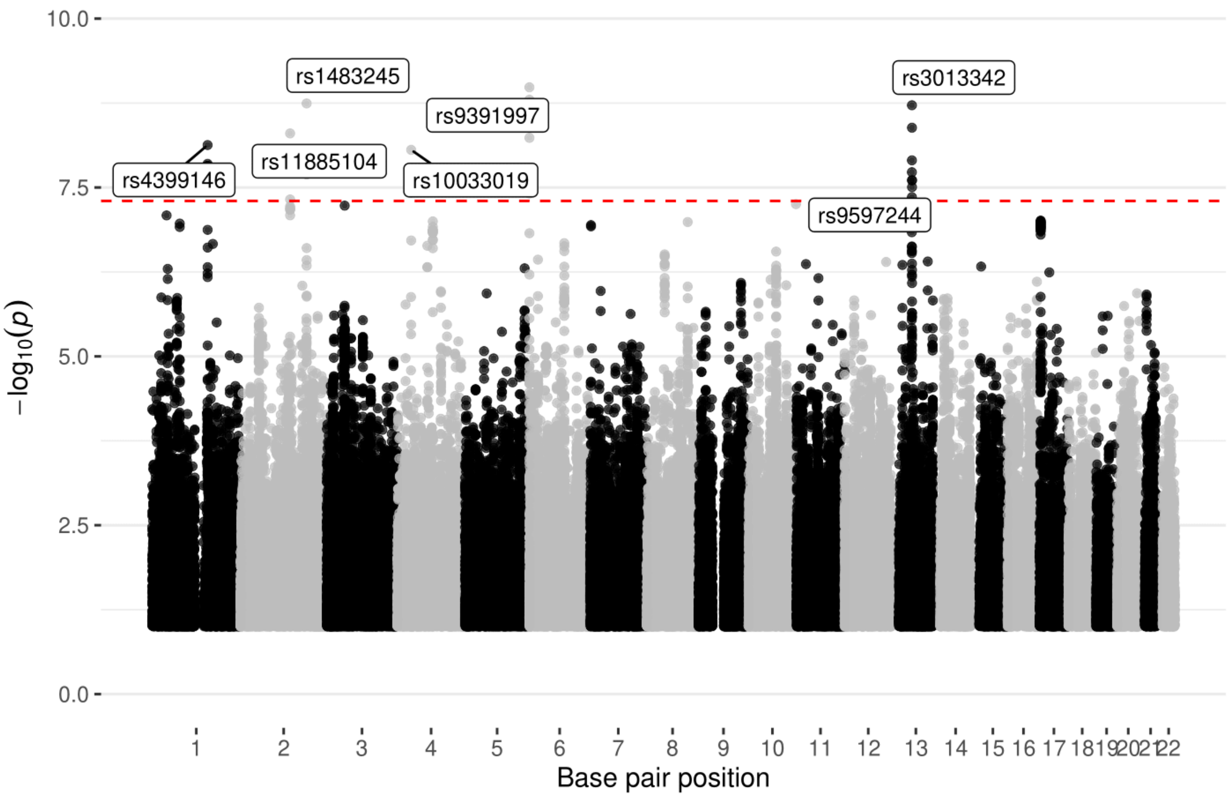

Figure S2: **Manhattan plot for the GWAS on the inverse probability weights.** The p-values are displayed on the y-axis on a $-log_{10}$ scale. The red line marks the genomewide significant threshold ($P = 5 \times 10^{-8}$). Approximately independent genomewide significant SNPs were assessed through clumping ($R^2 = 0.1$, window size 250kb). These top hits are annotated.

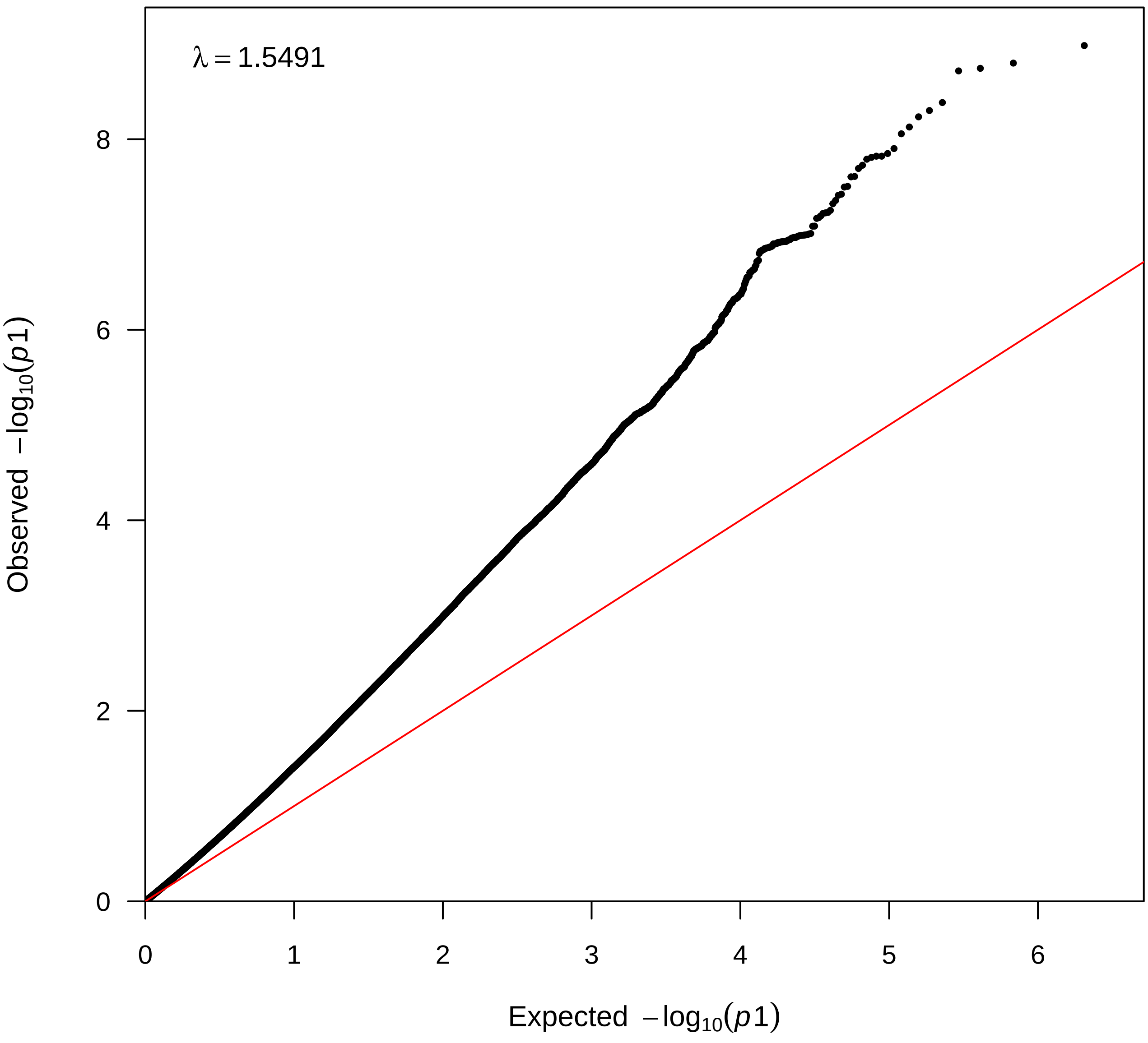

Figure S3: Quantile-quantile plot for the GWAS on the inverse probability weights. λ refers to the genomic inflation factor.

Figure S4: QQ plots of p-values which test for the difference between SNP associations estimated by GWAS and WGWAS for various phenotypes, as estimated by a Hausman test (see Methods). λ refers to the genomic inflation factor.

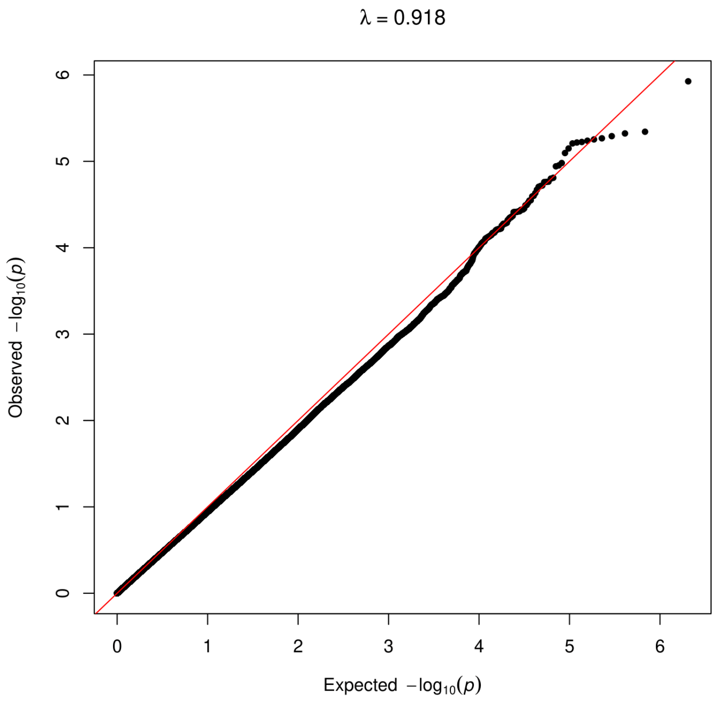

(a) AgeFirstBirth

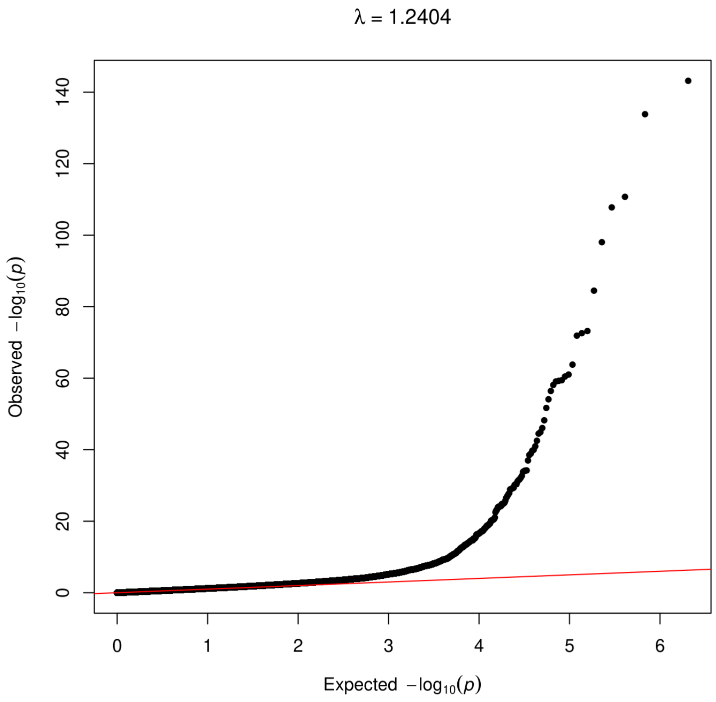

(b) Breast Cancer

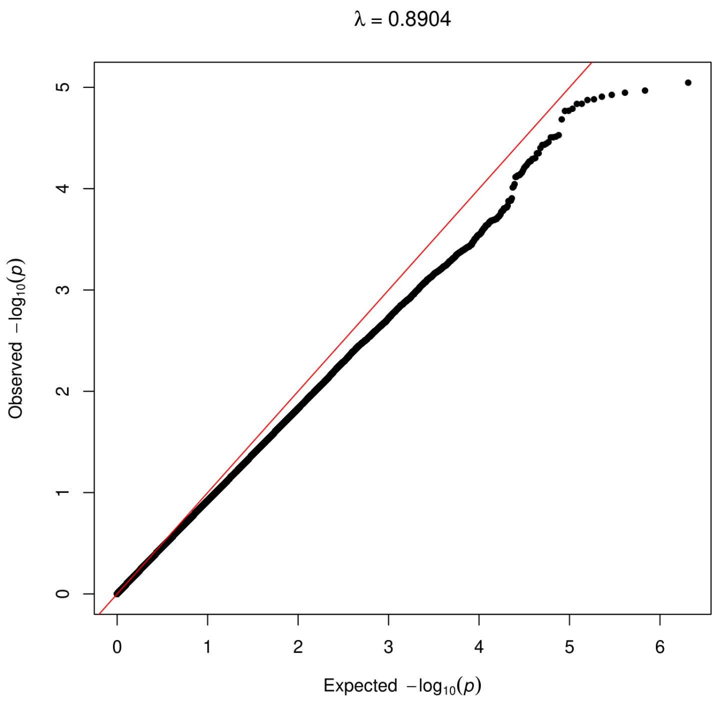

(c) BMI

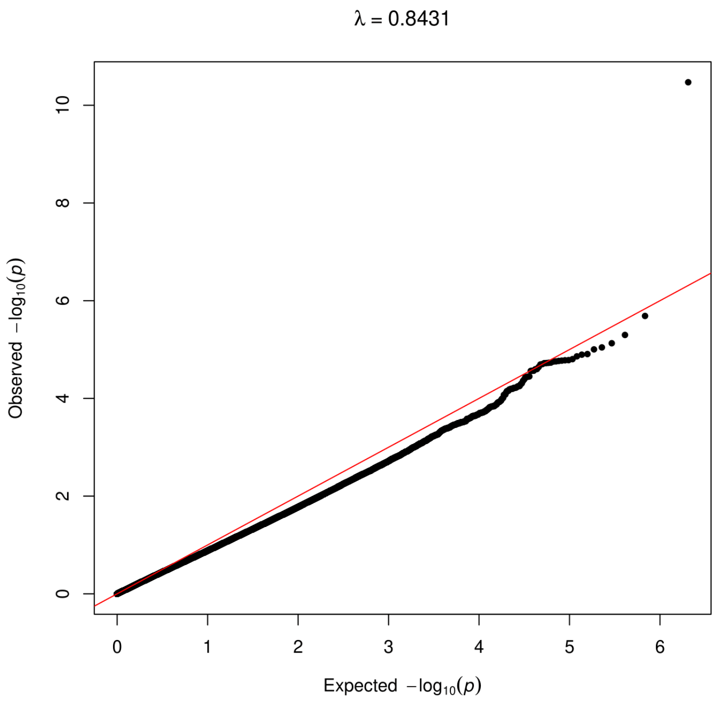

(d) Drinks Per Week

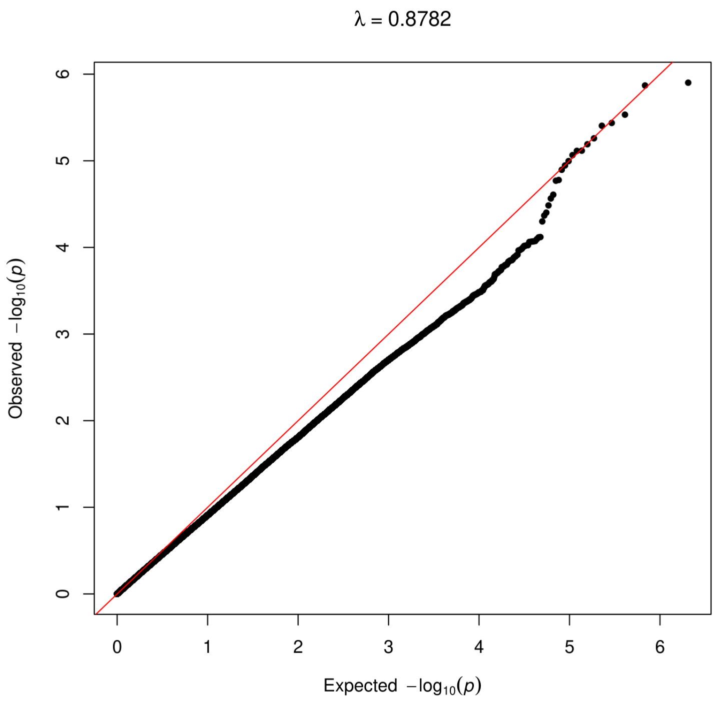

(e) Self-rated health

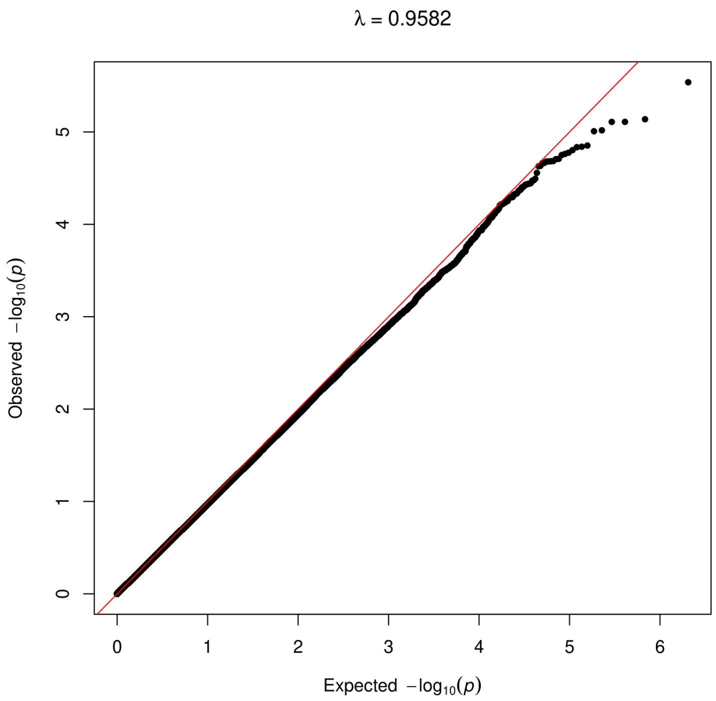

(f) Height

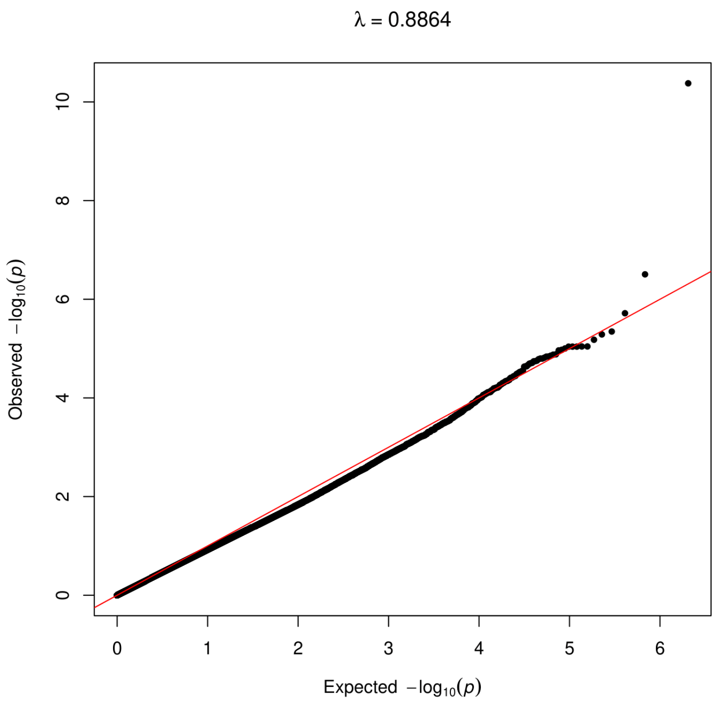

(g) Physical Activity

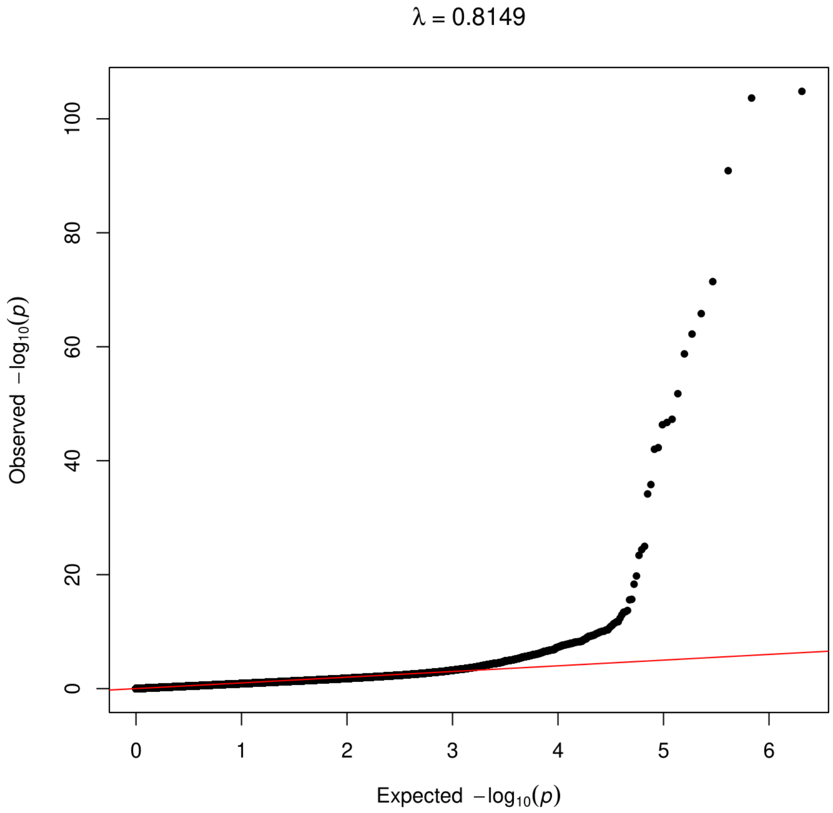

(h) Type 1 Diabetes

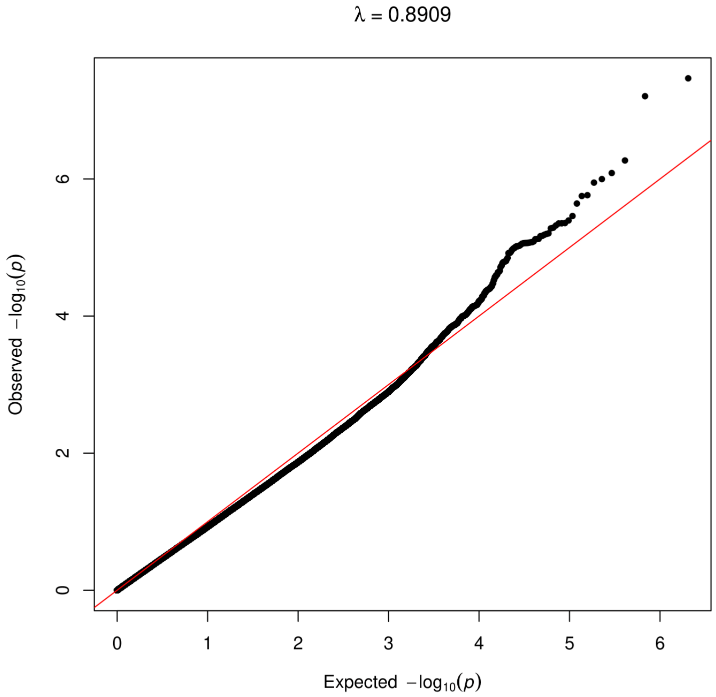

(i) Severe Obesity

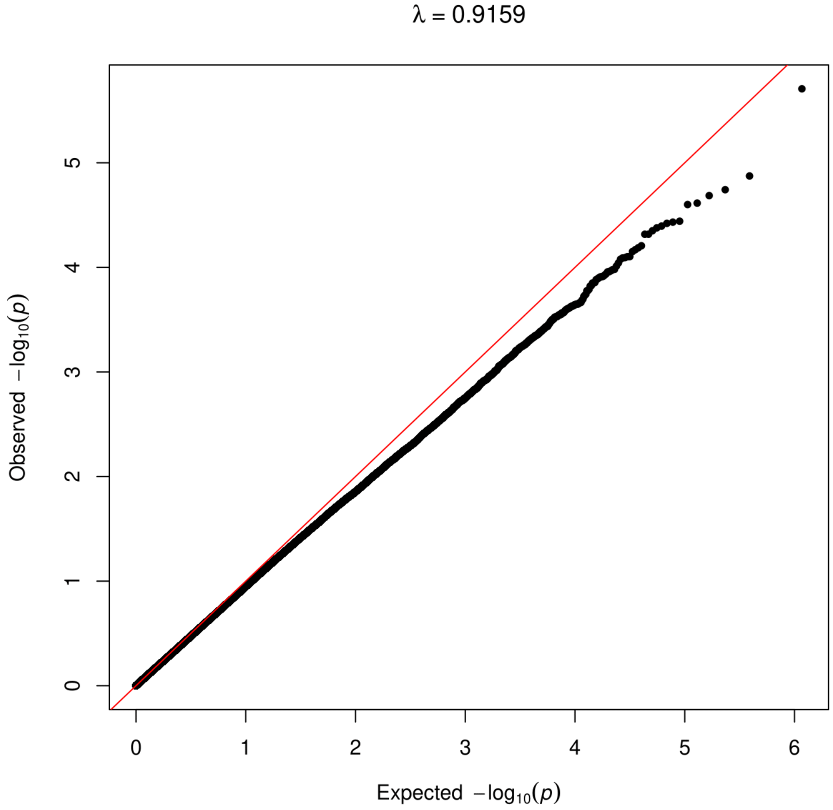

(j) Years of Education

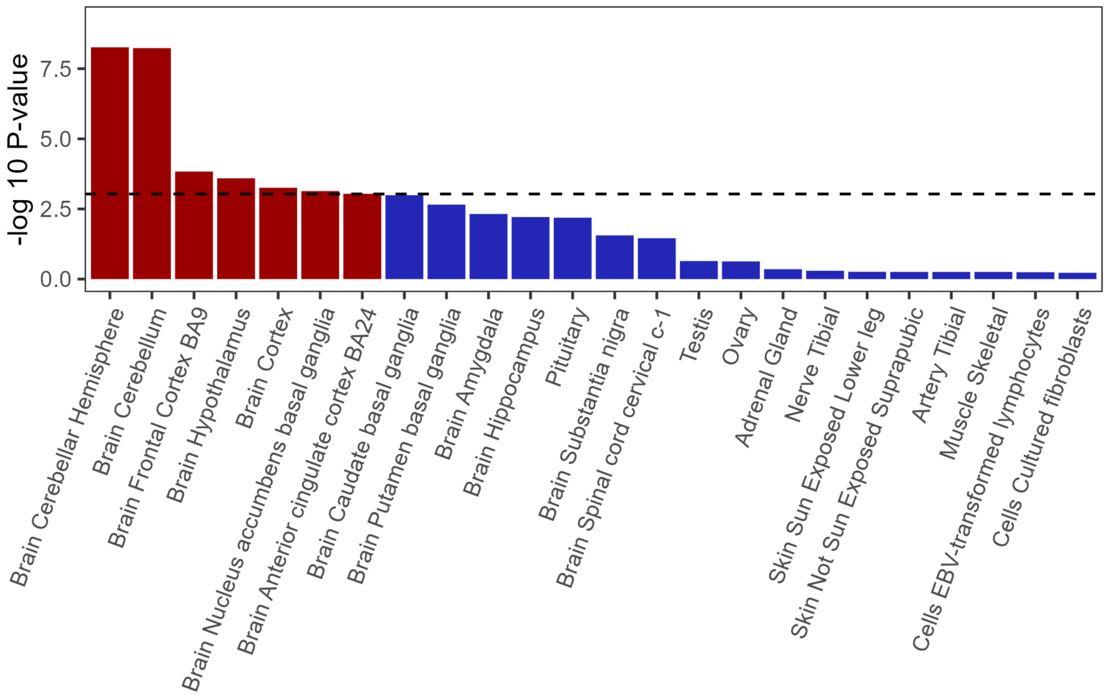

(a) AFB GWAS

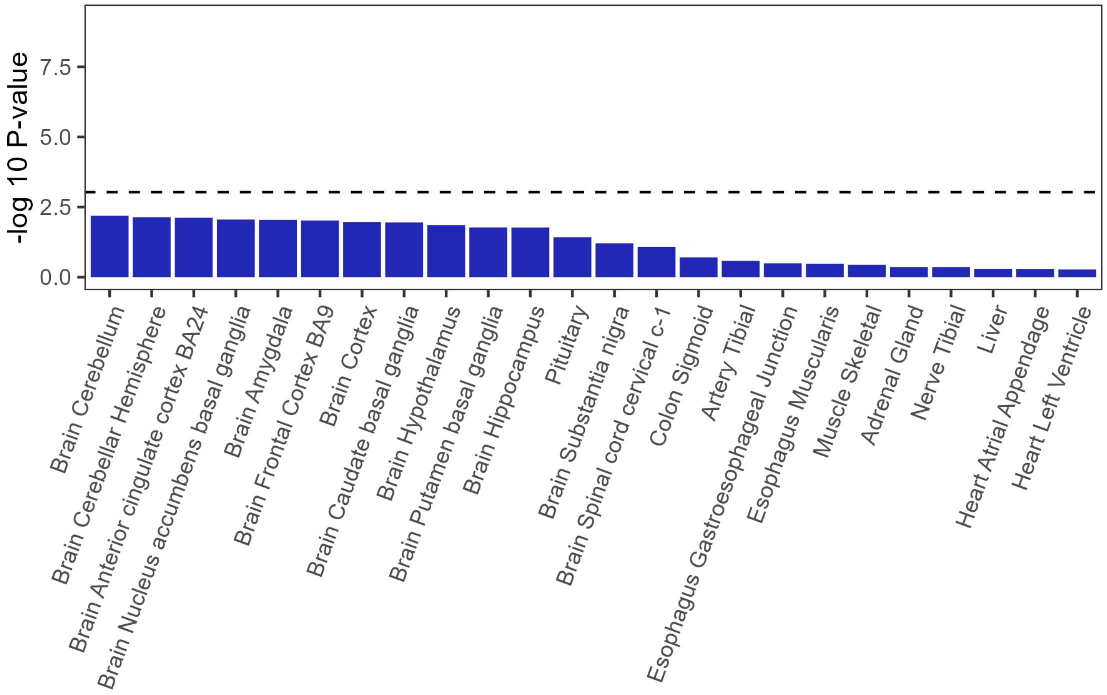

(b) AFB WGWAS

Figure S5: Gene tissue expression analysis estimated through MAGMA (implemented in FUMA) using GWAS/WGWAS results for Age at first birth

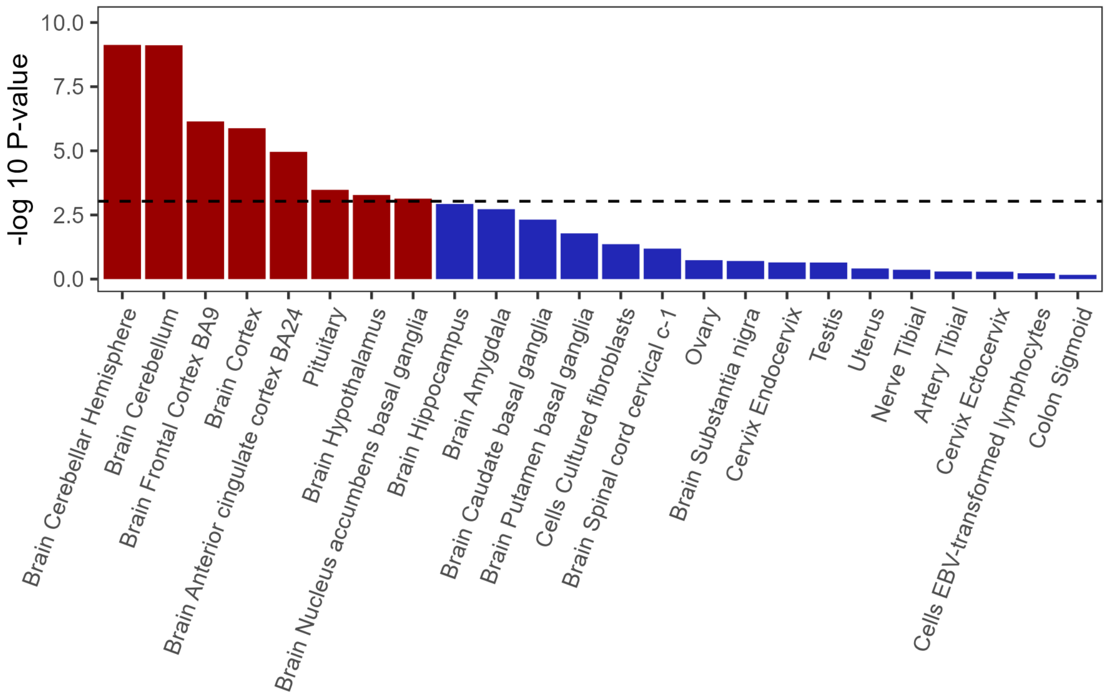

(a) BMI GWAS

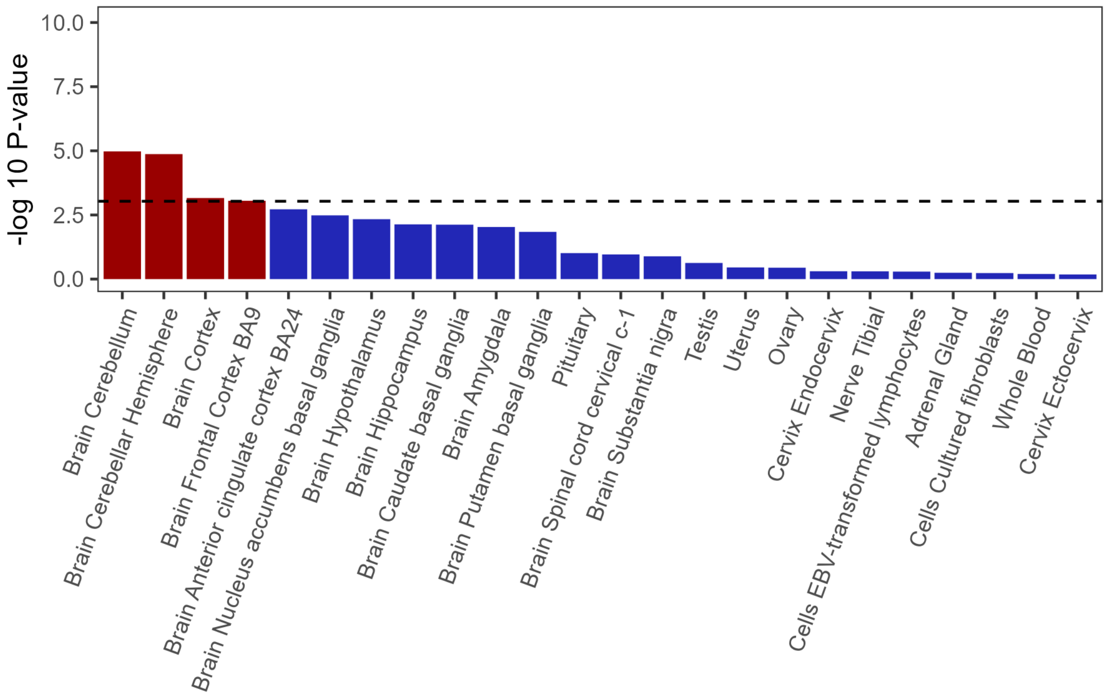

(b) BMI WGWAS

Figure S6: Gene tissue expression analysis estimated through MAGMA (implemented in FUMA) using GWAS/WGWAS results for BMI

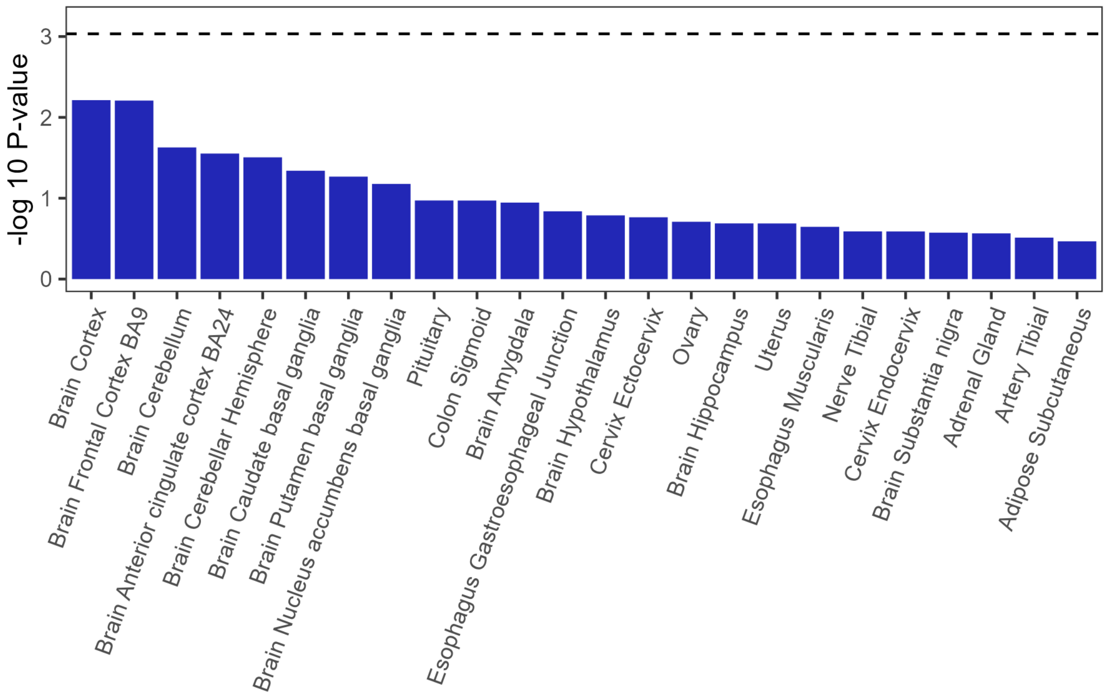

(a) Drinks Per Week GWAS

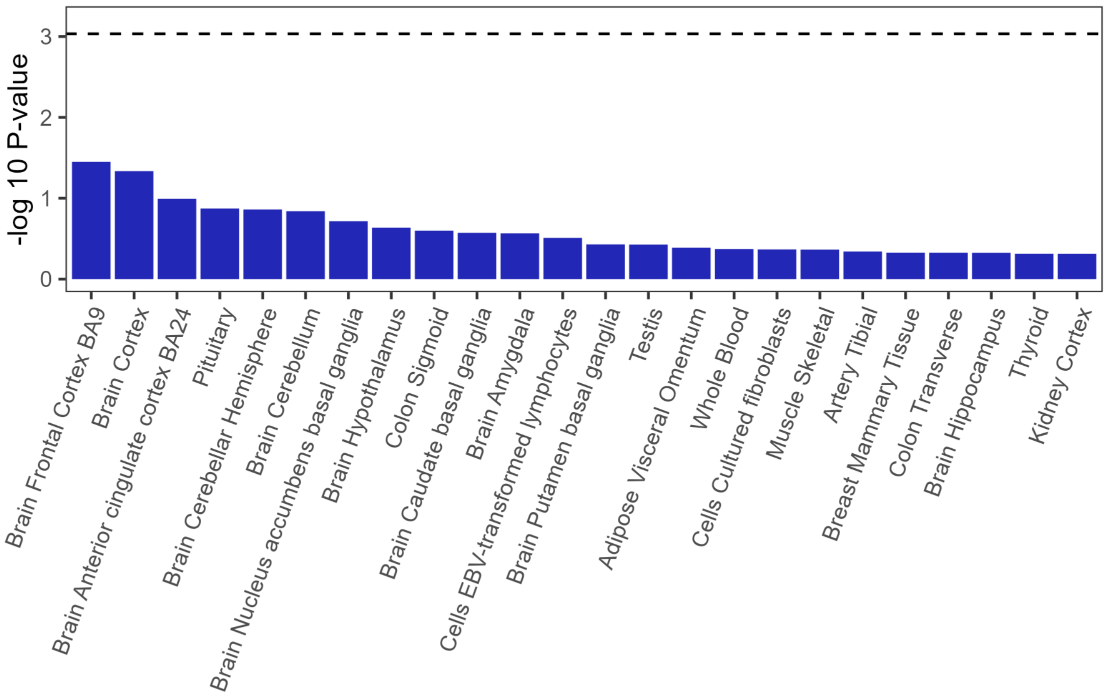

(b) Drinks Per Week WGWAS

Figure S7: Gene tissue expression analysis estimated through MAGMA (implemented in FUMA) using GWAS/WGWAS results for Drinks per week

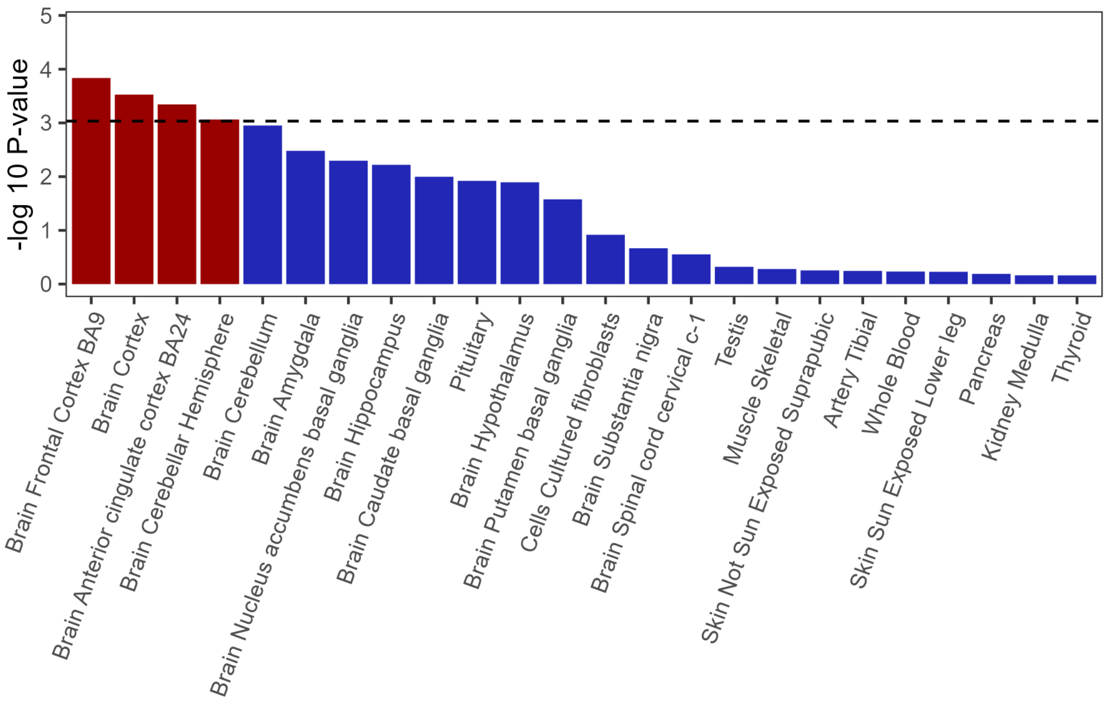

(a) Self-reported health GWAS

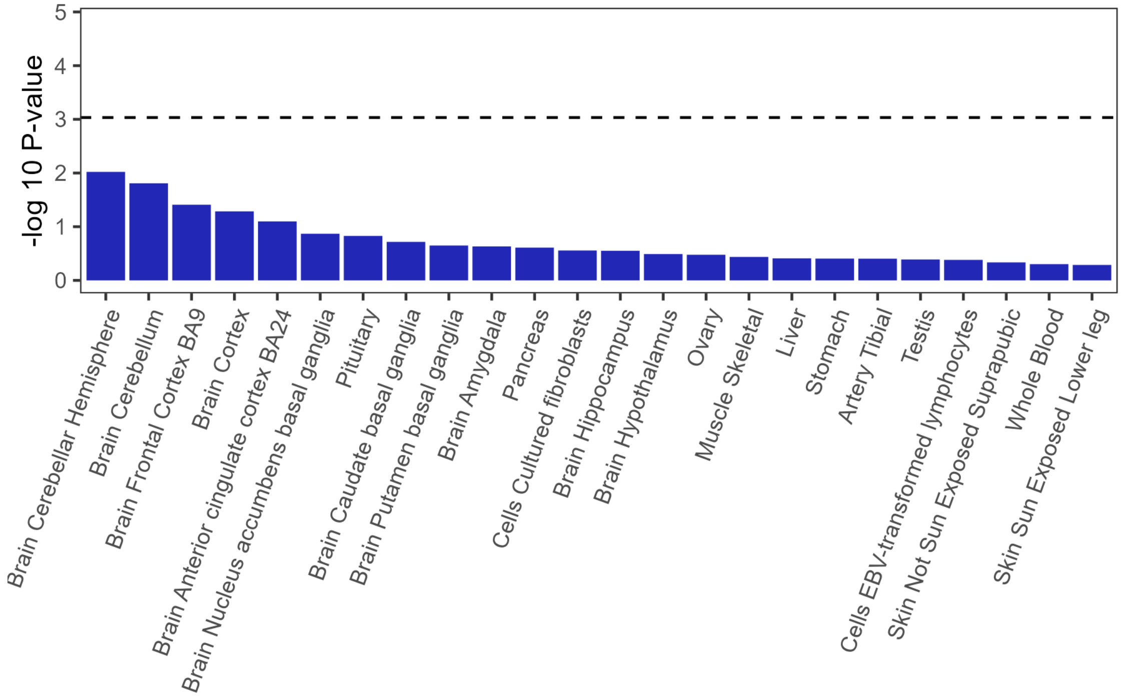

(b) Self-reported health WGWAS

Figure S8: Gene tissue expression analysis estimated through MAGMA (implemented in FUMA) using GWAS/WGWAS results for Self-rated health

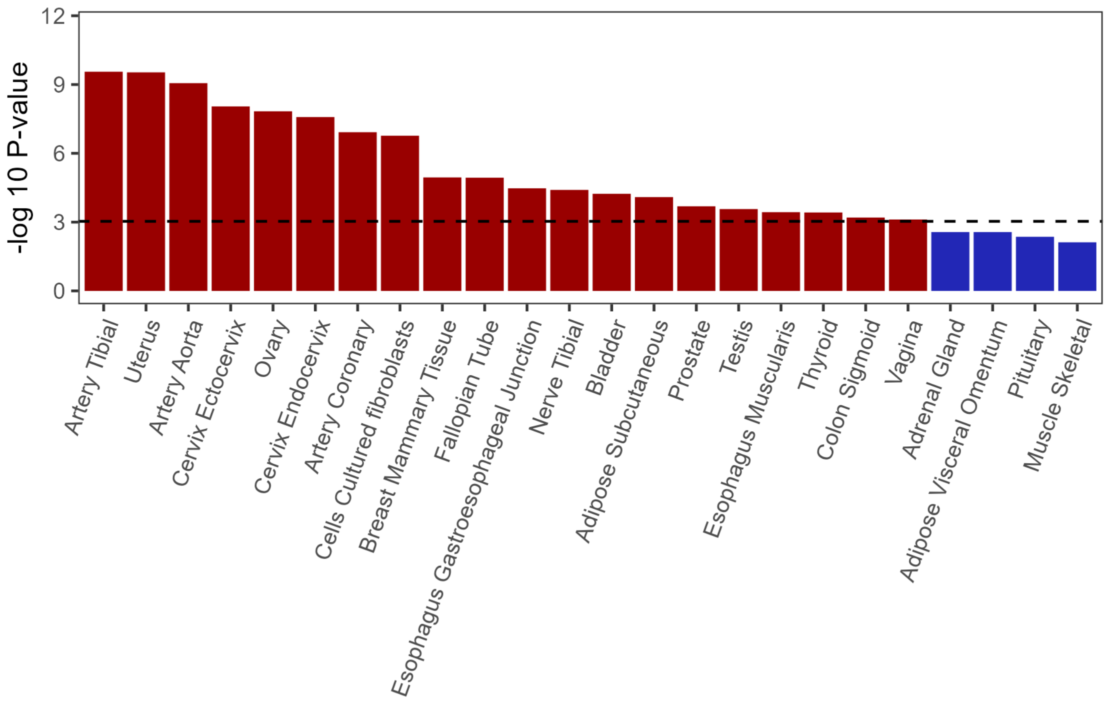

(a) Height GWAS

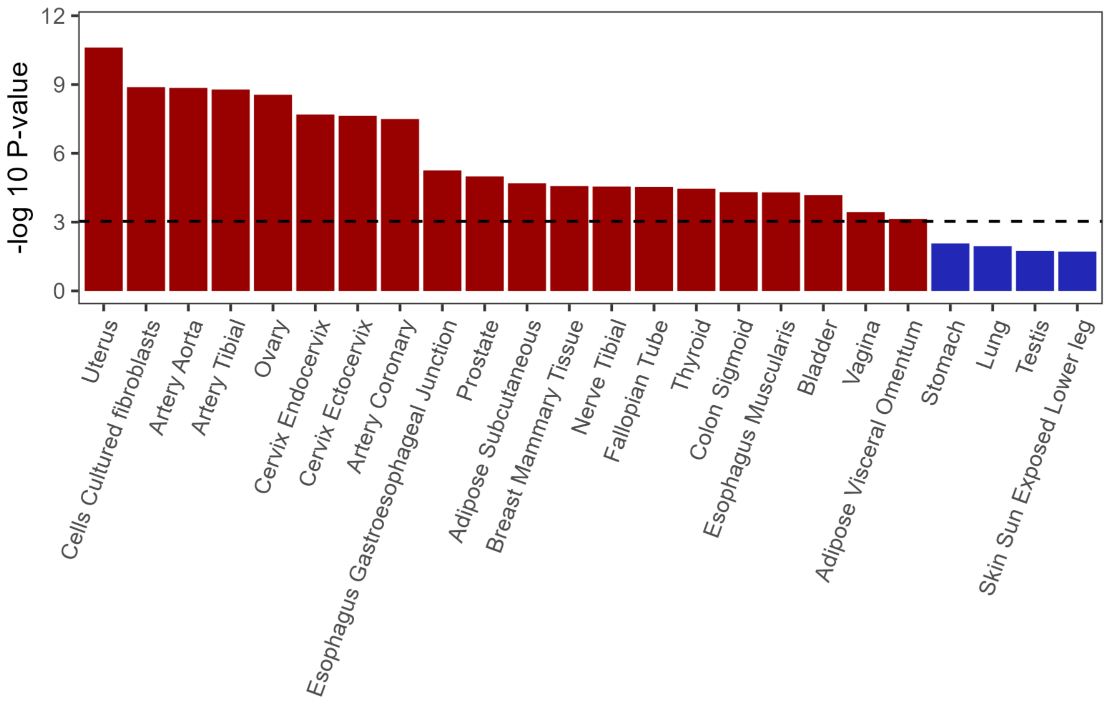

(b) Height WGWAS

Figure S9: Gene tissue expression analysis estimated through MAGMA (implemented in FUMA) using GWAS/WGWAS results for Height

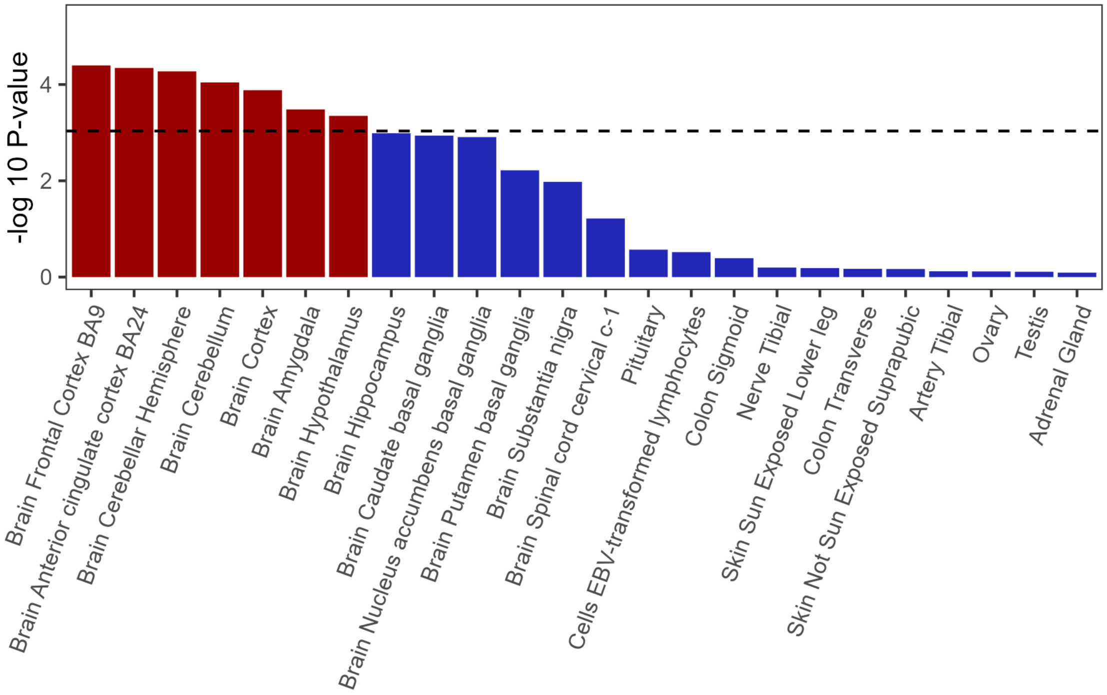

(a) Physical Activity GWAS

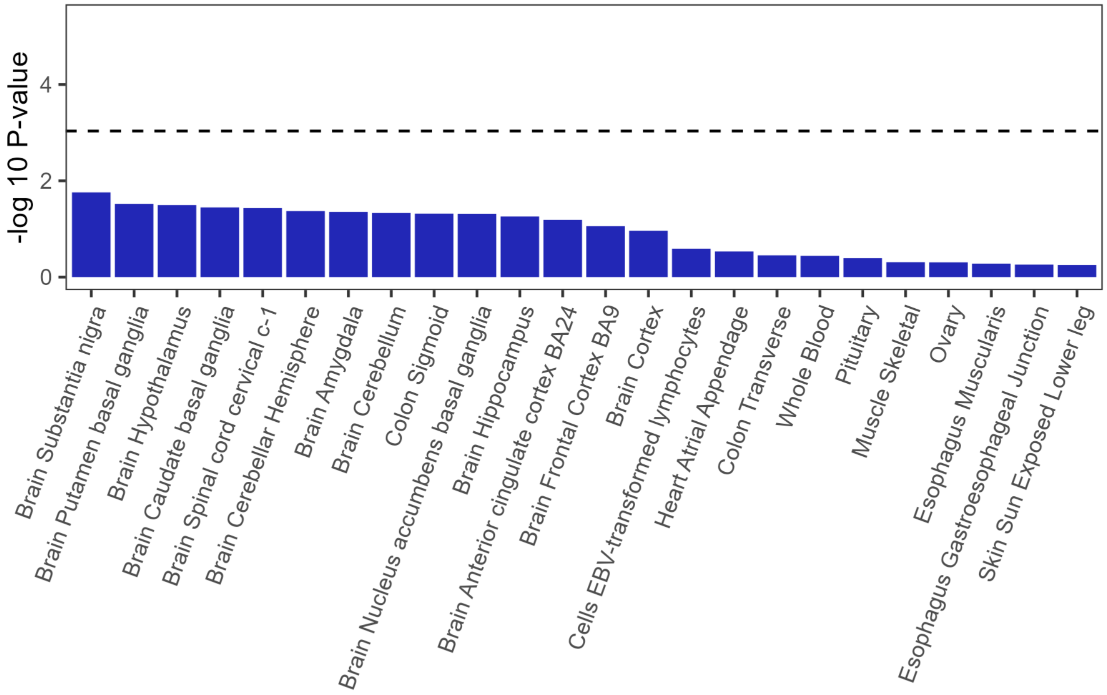

(b) Physical Activity WGWAS

Figure S10: Gene tissue expression analysis estimated through MAGMA (implemented in FUMA) using GWAS/WGWAS results for Physical Activity

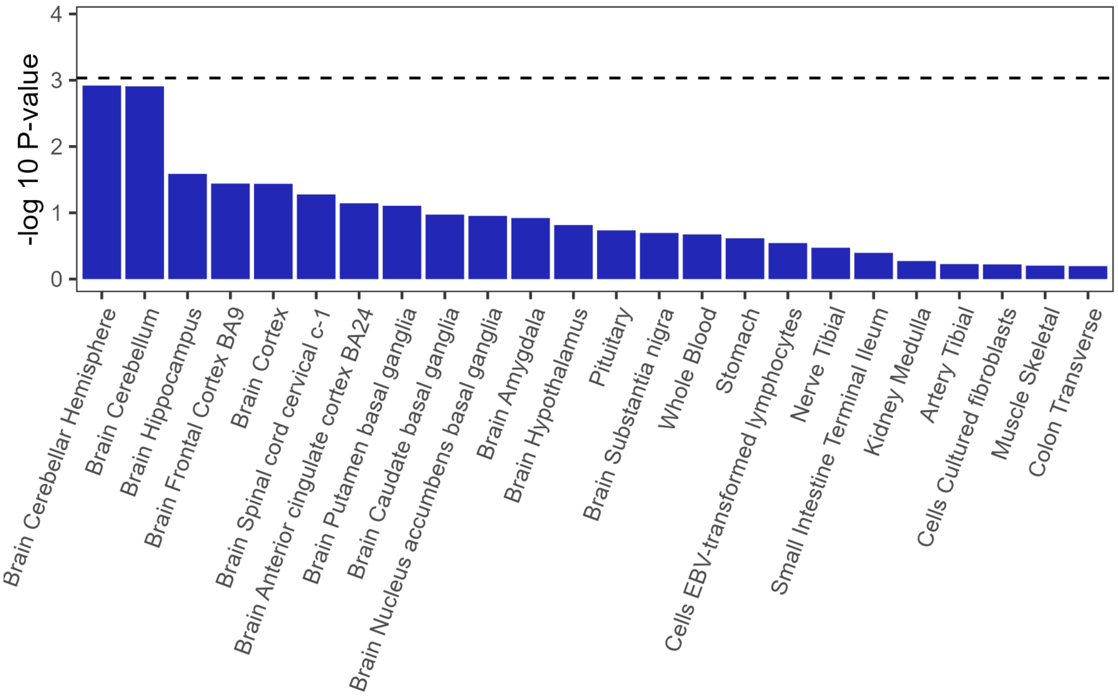

(a) Severe Obesity GWAS

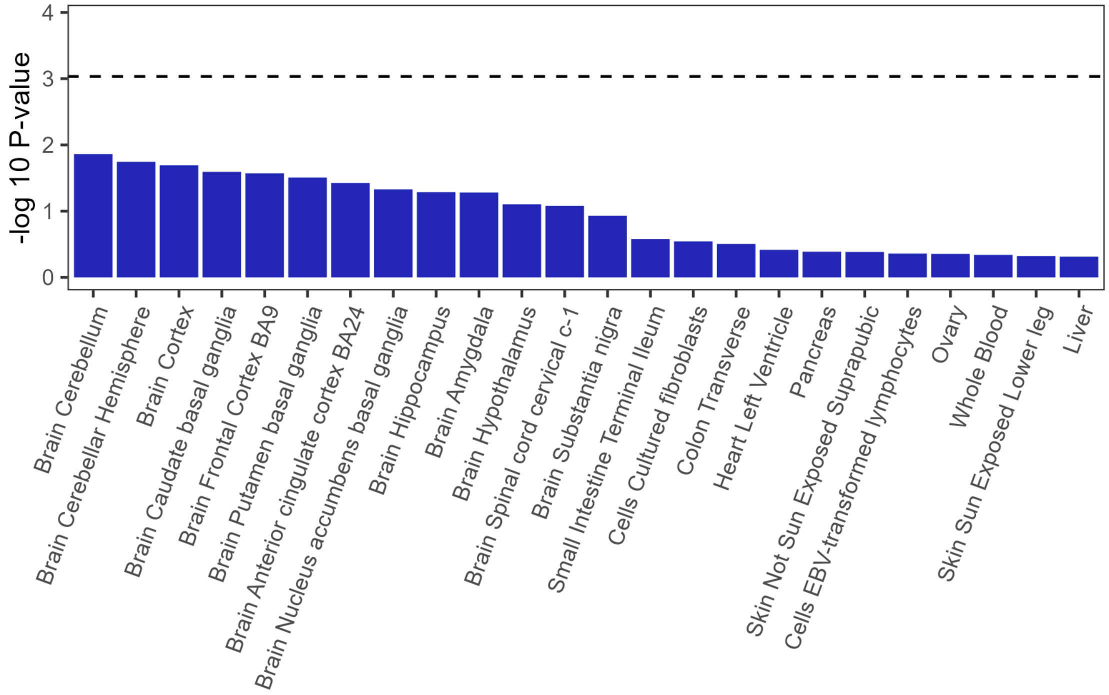

(b) Severe Obesity WGWAS

Figure S11: Gene tissue expression analysis estimated through MAGMA (implemented in FUMA) using GWAS/WGWAS results for Severe obesity

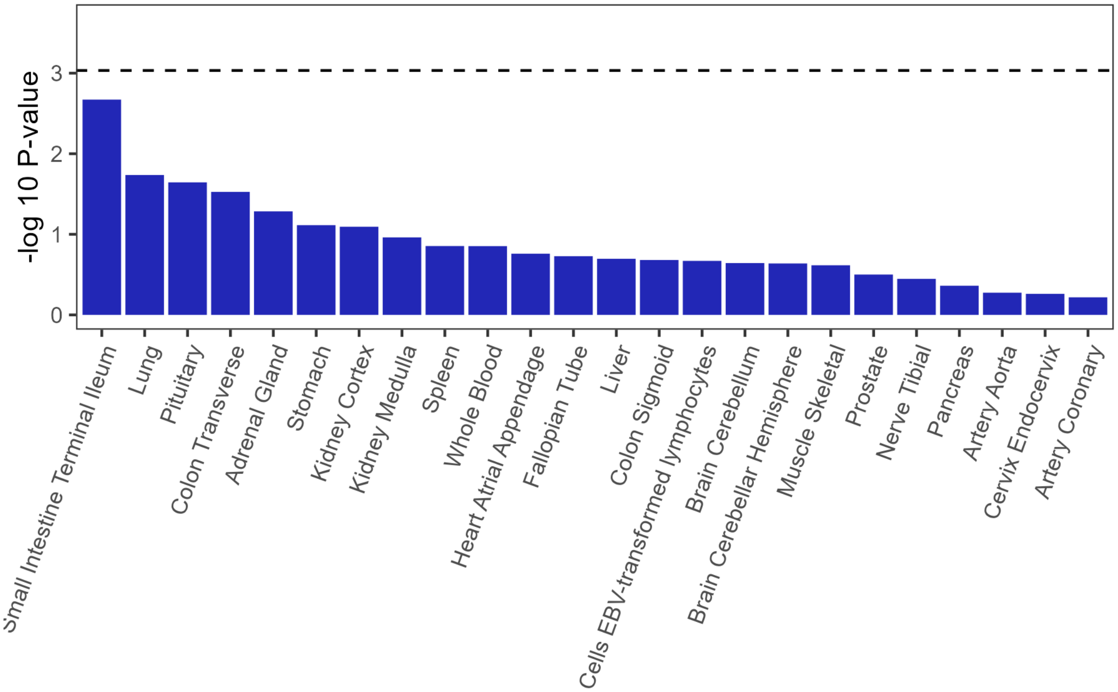

(a) Type 1 Diabetes GWAS

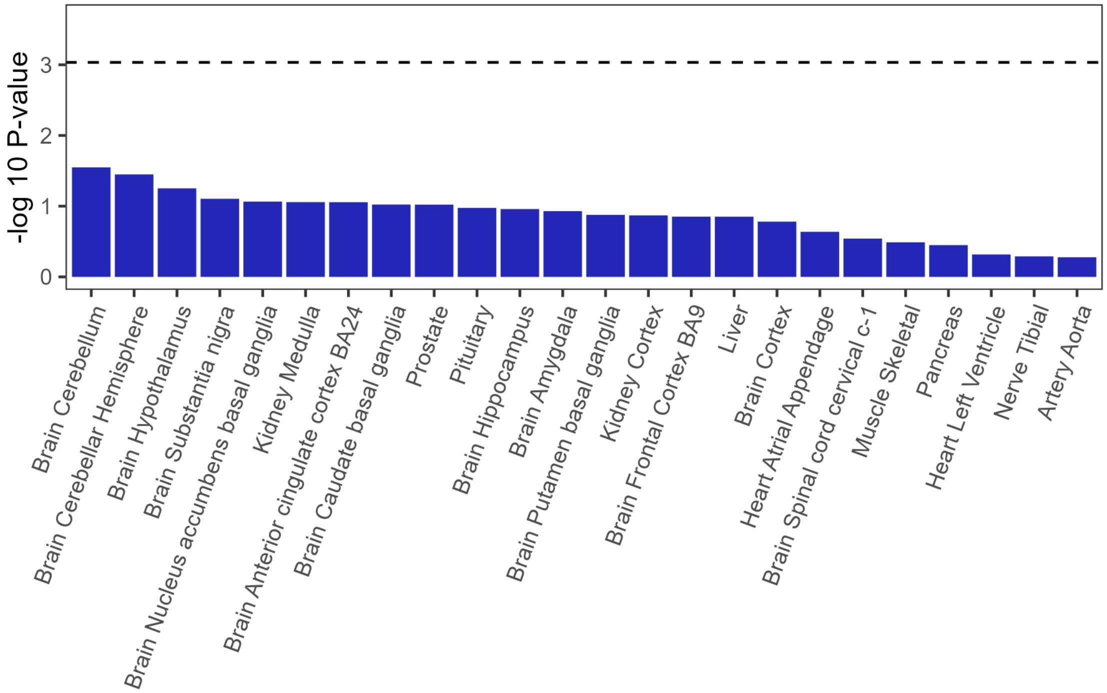

(b) Type 1 Diabetes WGWAS

Figure S12: Gene tissue expression analysis estimated through MAGMA (implemented in FUMA) using GWAS/WGWAS results for Type 1 diabetes

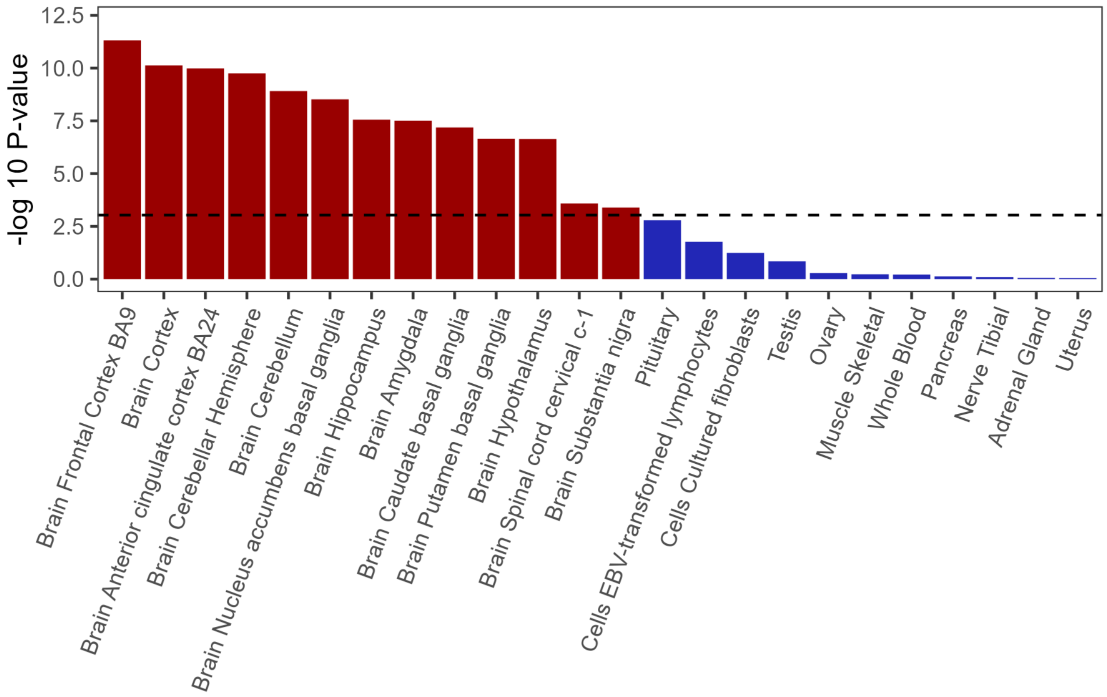

(a) Years of Education GWAS

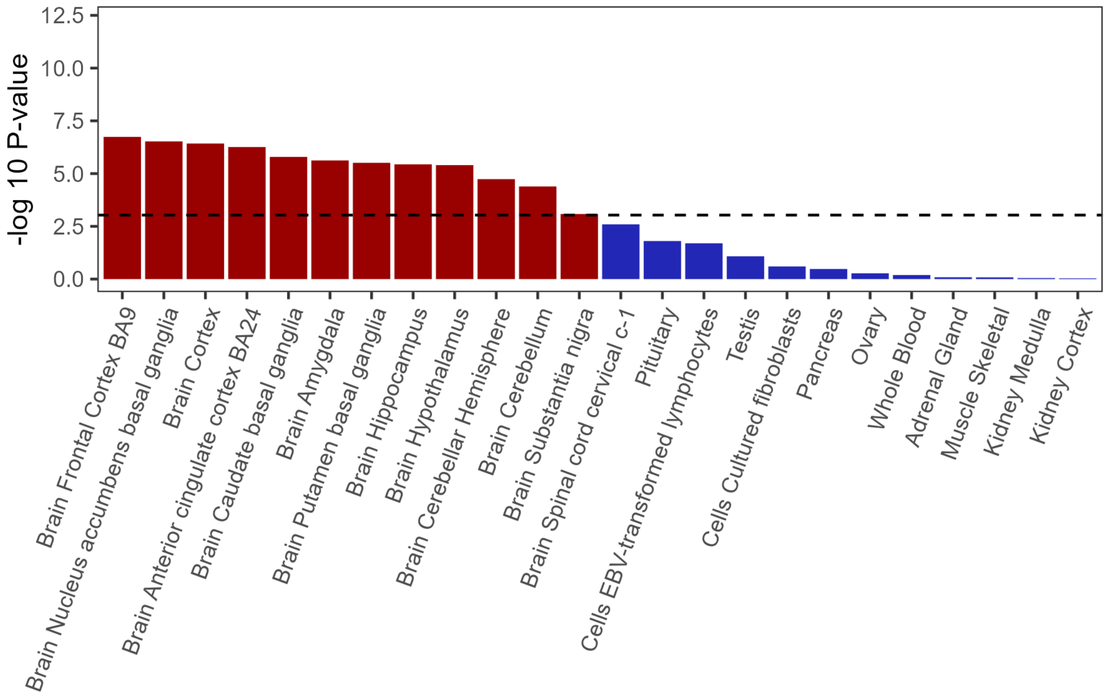

(b) Years of Education WGWAS

Figure S13: Gene tissue expression analysis estimated through MAGMA (implemented in FUMA) using GWAS/WGWAS results for Years of Education

Figure S14: **Zoomed in Manhattan plots of GWAS associations with the UKB inverse probability weights.** Here, we focus on *all* SNPs that are in linkage disequilibrium ($R^2 > 0.1$, 500 kb) with one of the 7 identified lead SNPs for the IP weights ($P < 5 \cdot 10^{-8}$). SNPs that have been found to significantly associate with other traits as found in the GWAS catalog ($P < 5 \cdot 10^{-8}$) are annotated with this trait. The dotted horizontal line shows the genomewide significance level on the negative log scale. Each dot in the plot shows the p-value and base pair position of the association between a SNP (in linkage disequilibrium with the lead SNP) and the IP weights. The lead SNP is depicted by the cross. Each dot's color reflects the level of linkage disequilibrium with the lead SNP, as measured by the R^2.

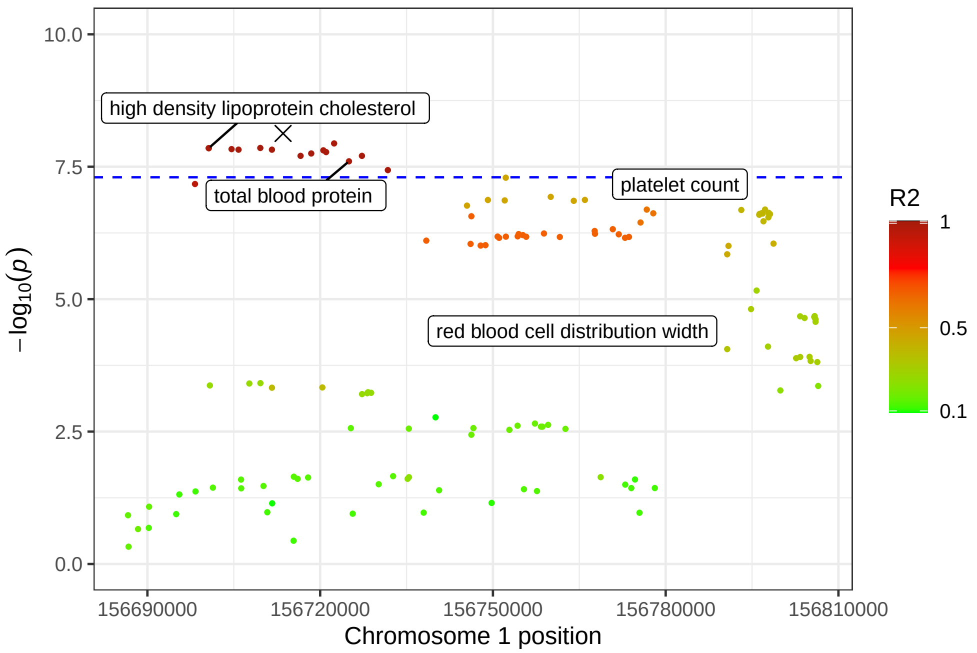

(a) lead SNP rs4399146

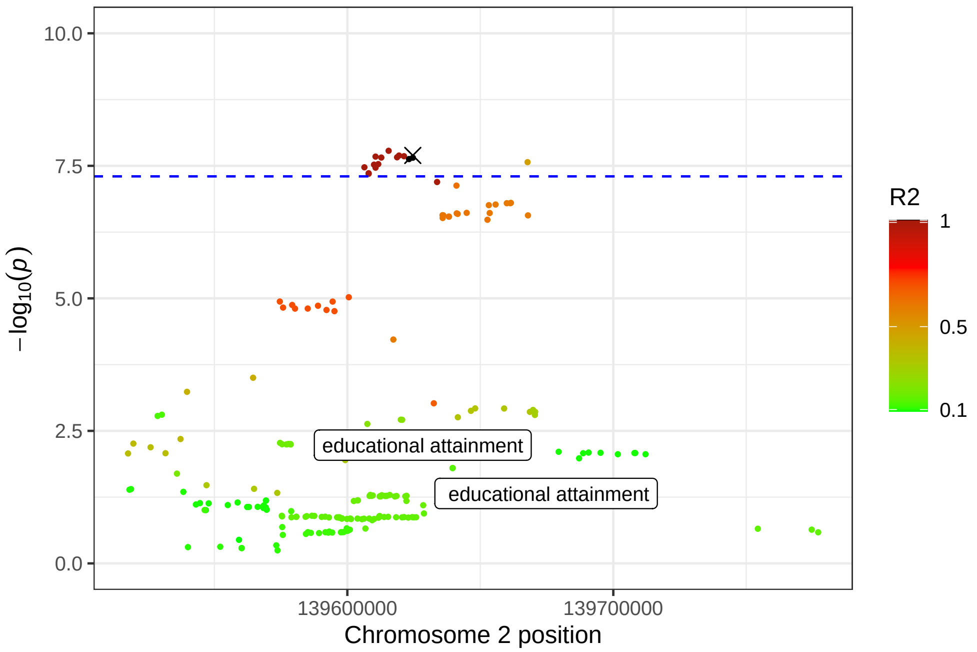

(b) lead SNP rs11885104

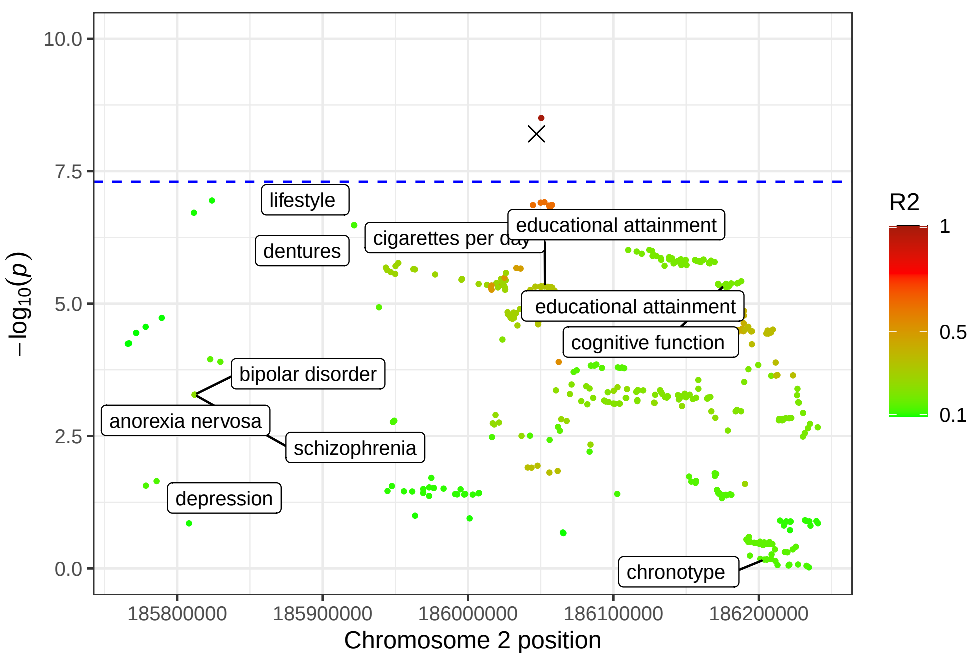

(c) lead SNP rs1483245

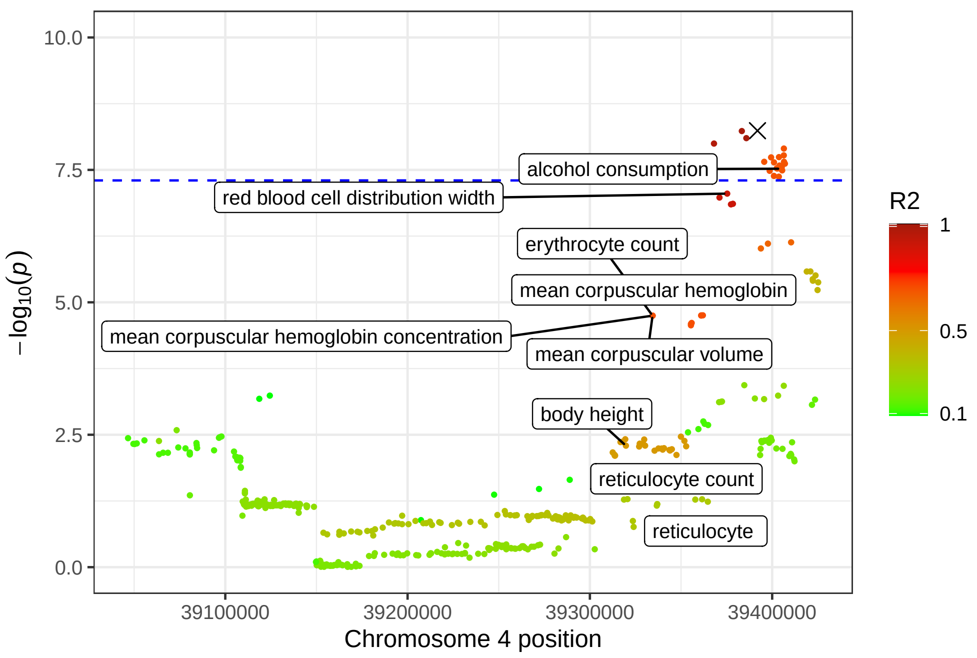

(d) lead SNP rs10033019

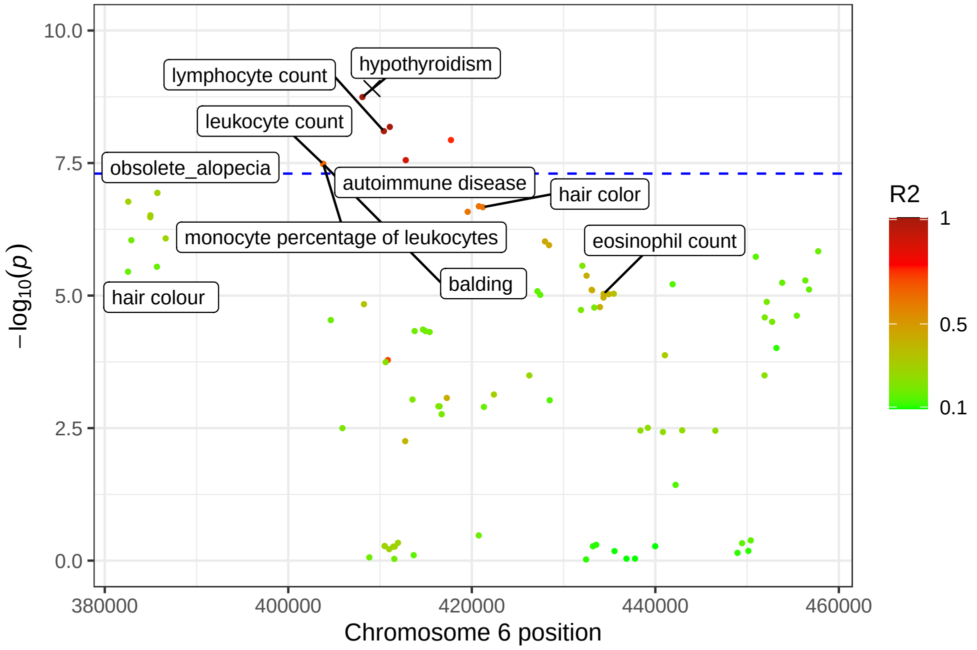

(e) lead SNP rs9391997

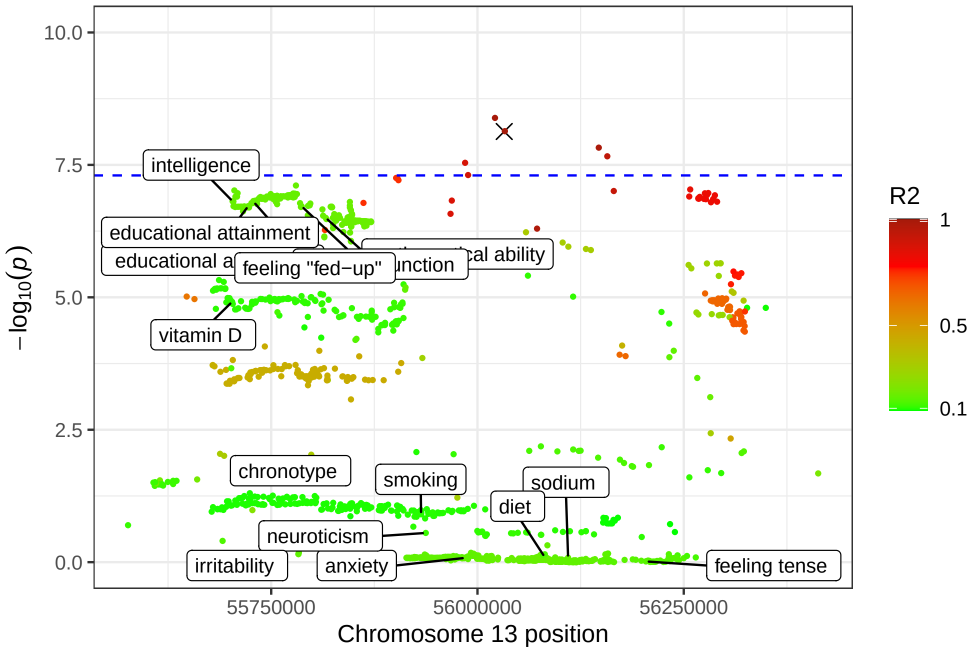

(f) lead SNP rs3013342

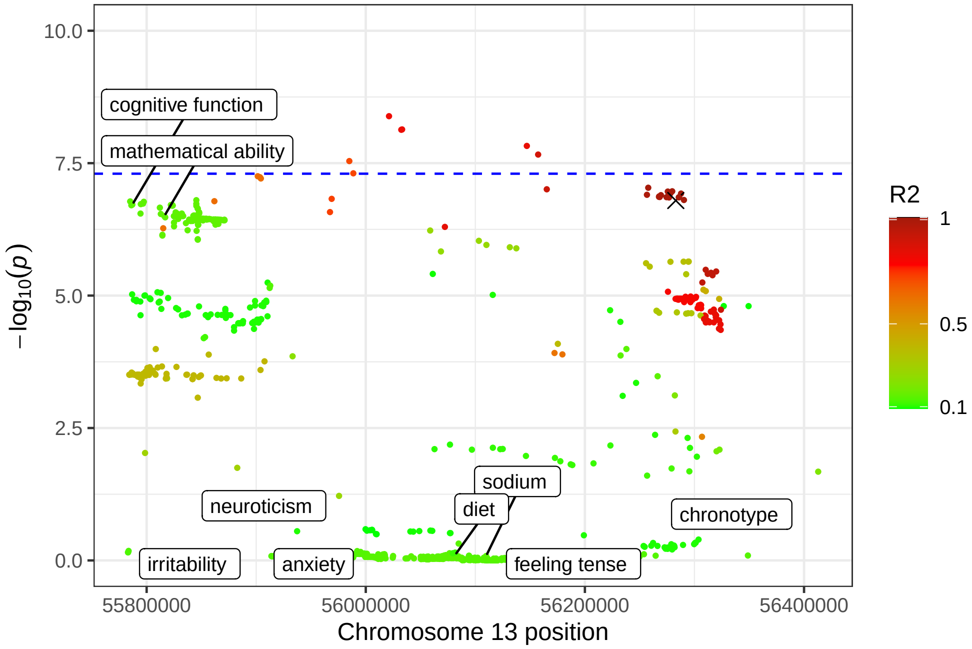

(g) lead SNP rs9597244

Figure S15: **Zoomed in Manhattan plots of WGWAS associations with type 1 diabetes.** Here, we focus on *all* SNPs that are in linkage disequilibrium ($R^2 > 0.1$, 500 kb) with one of the 3 newly identified lead SNPs for type 1 diabetes as found in WGWAS ($P < 5 \cdot 10^{-8}$ in WGWAS *and* $P_H < 5 \cdot 10^{-8}$). SNPs that have been found to significantly associate with other traits as found in the GWAS catalog ($P < 5 \cdot 10^{-5}$) are annotated with this trait. The dotted horizontal line shows the genomewide significance level on the negative log scale. Each dot in the plot shows the p-value and base pair position of the association between a SNP (in linkage disequilibrium with the lead SNP) and type 1 diabetes. The lead SNP is depicted as the cross. Each dot is colored by the level of linkage disequilibrium with this lead SNP, as measured by the R^2.

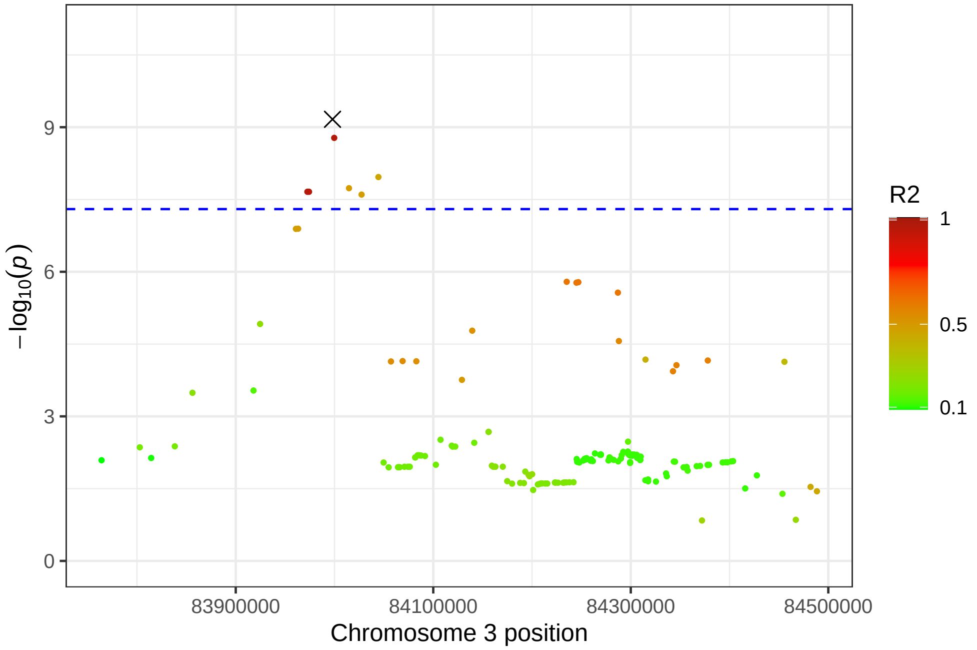

(a) lead SNP rs9861858

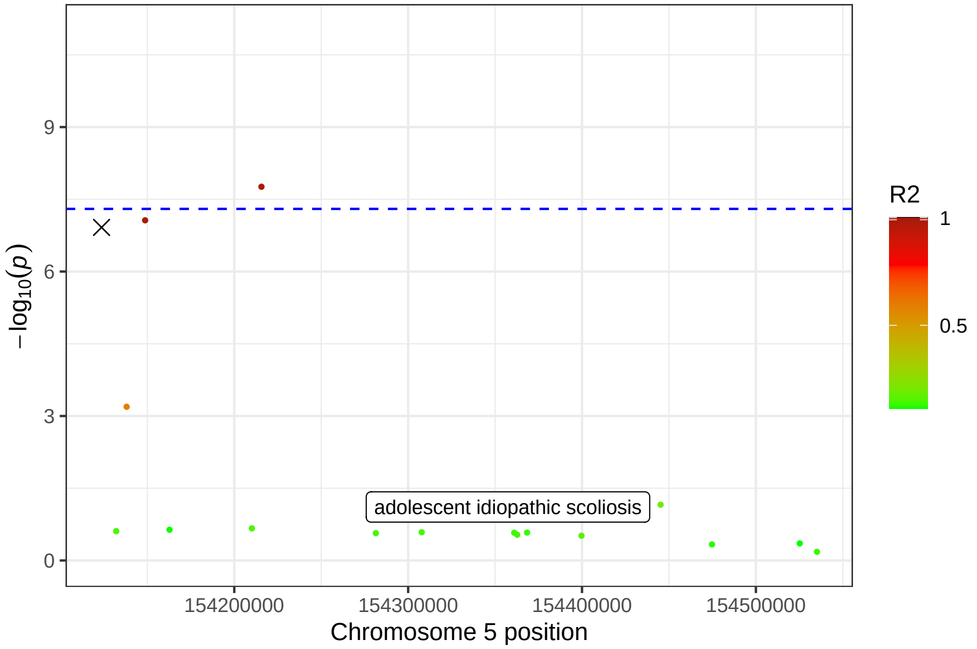

(b) lead SNP rs12522568

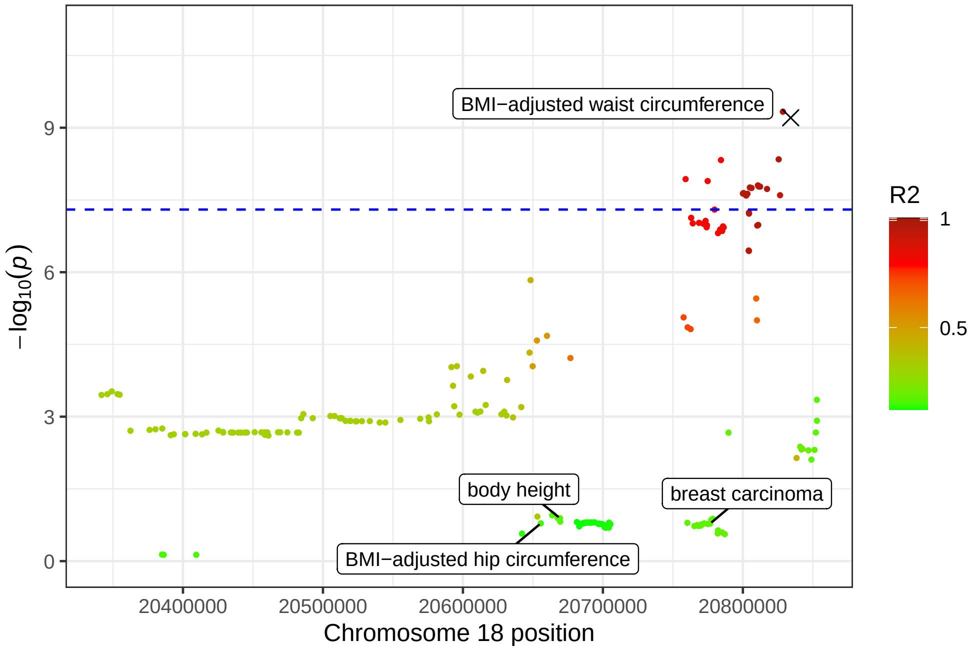

(c) lead SNP rs17186868

Figure S16: **Zoomed in Manhattan plot of WGWAS associations with breast cancer.** Here, we focus on *all* SNPs that are in linkage disequilibrium ($R^2 > 0.1$, 500 kb) with the newly identified lead SNP rs2306412 as found in WGWAS ($P < 5 \cdot 10^{-8}$ in WGWAS *and* $P_H < 5 \cdot 10^{-8}$). None of these SNPs were found to significantly associate with other traits as found in the GWAS catalog ($P < 5 \cdot 10^{-5}$). The lead SNP is depicted as the cross. Each dot is colored by the level of linkage disequilibrium with this lead SNP, as measured by the R^2.

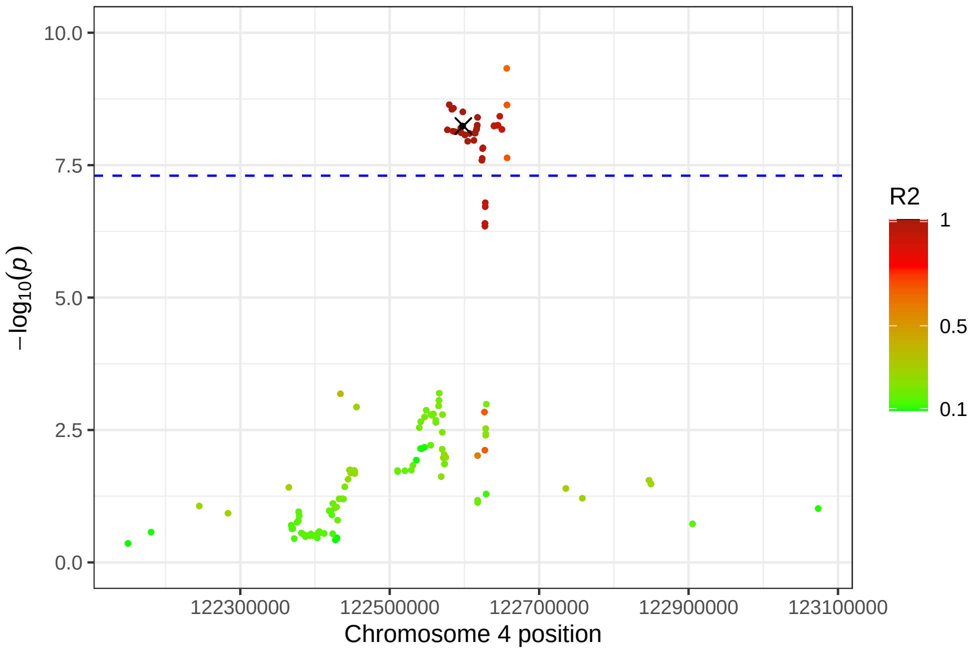

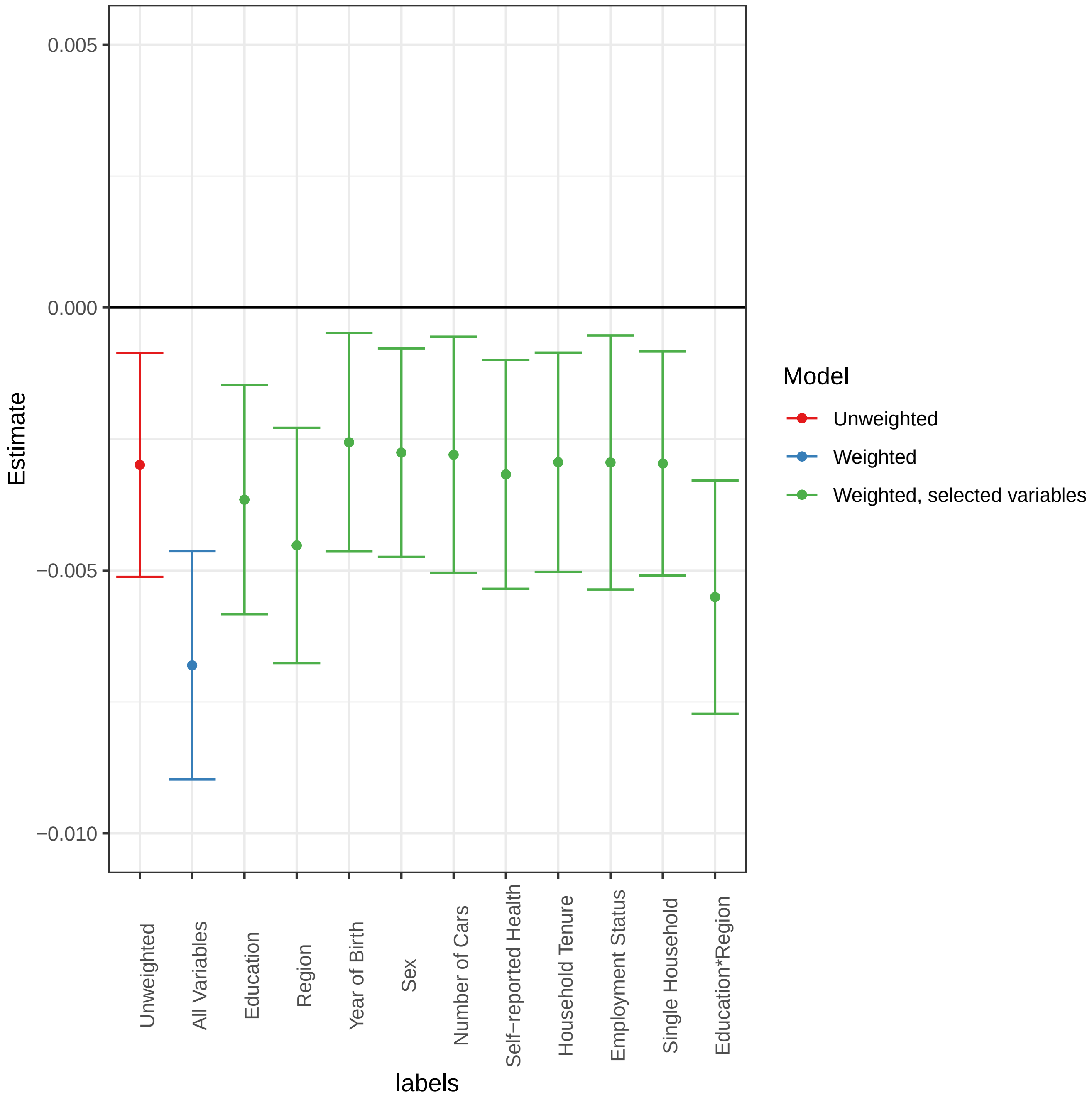

Figure S17: Association between *rs2306412* and breast cancer, unweighted, weighted using our original IP weights ("All Variables"), and weighted using weights that were estimated on a sparser set of variables (as denoted by "labels")."*" refers to a model where the two variables, and all their respective two-way interactions, are included in estimating the weights.

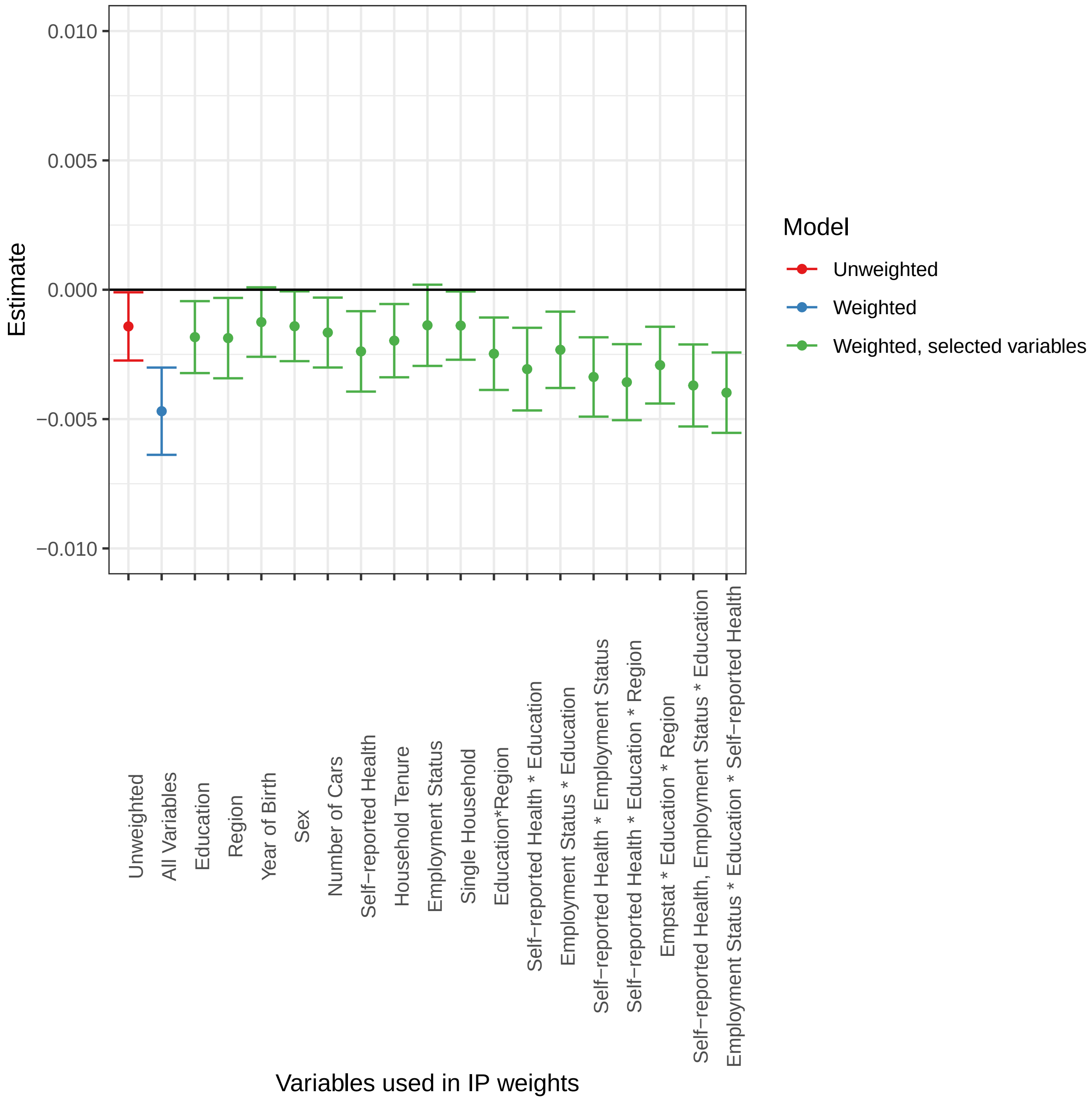

Figure S18: Association between *rs12522568* and type 1 diabetes, unweighted, weighted using our original IP weights ("All Variables"), and weighted using weights that were estimated on a sparser set of variables (as denoted by "labels")."*" refers to a model where the two variables, and all their respective two-way interactions, are included in estimating the weights.

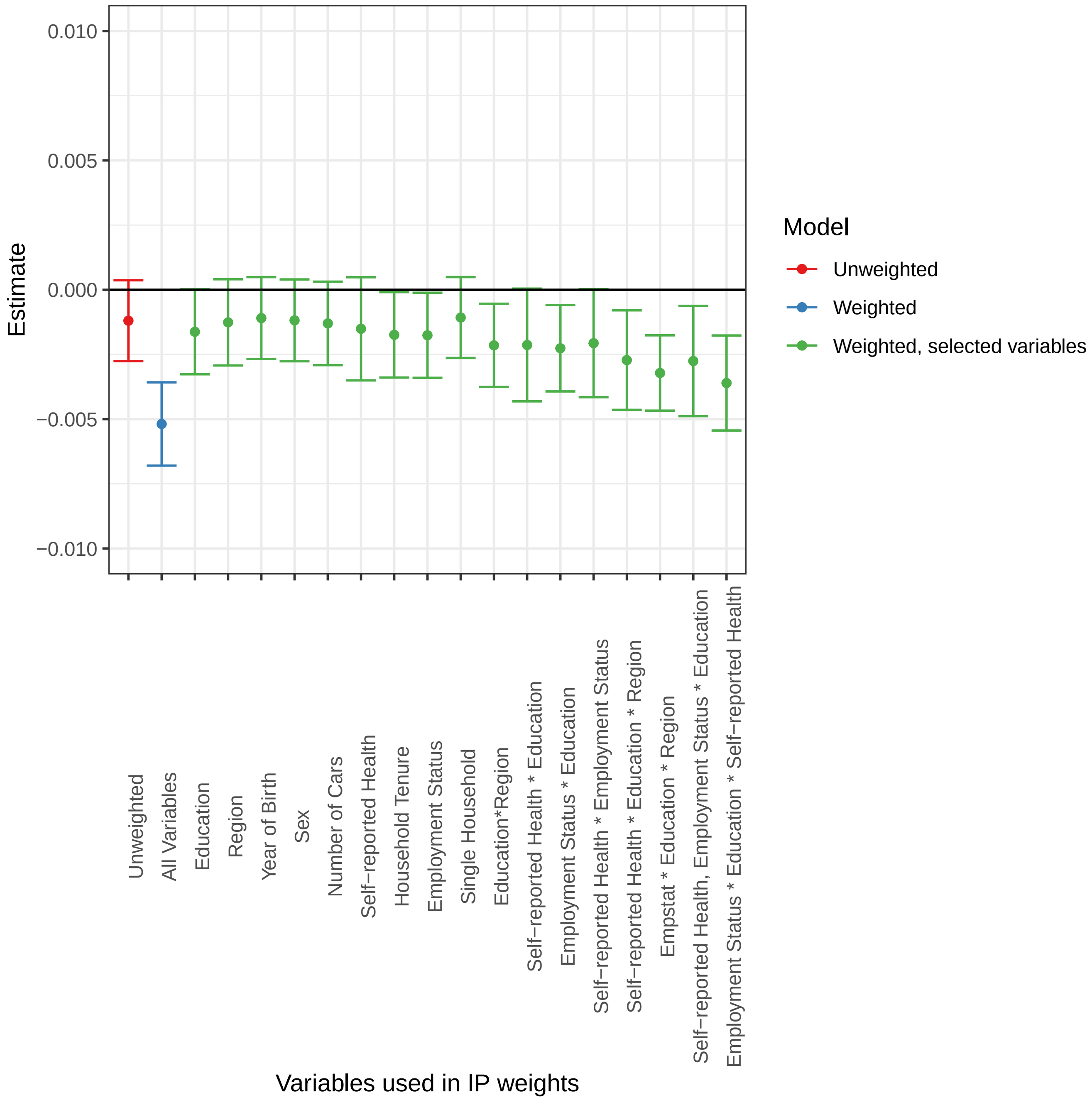

Figure S19: Association between *rs1718686* and type 1 diabetes, unweighted, weighted using our original IP weights ("All Variables"), and weighted using weights that were estimated on a sparser set of variables (as denoted by "labels")."*" refers to a model where the two variables, and all their respective two-way interactions, are included in estimating the weights.

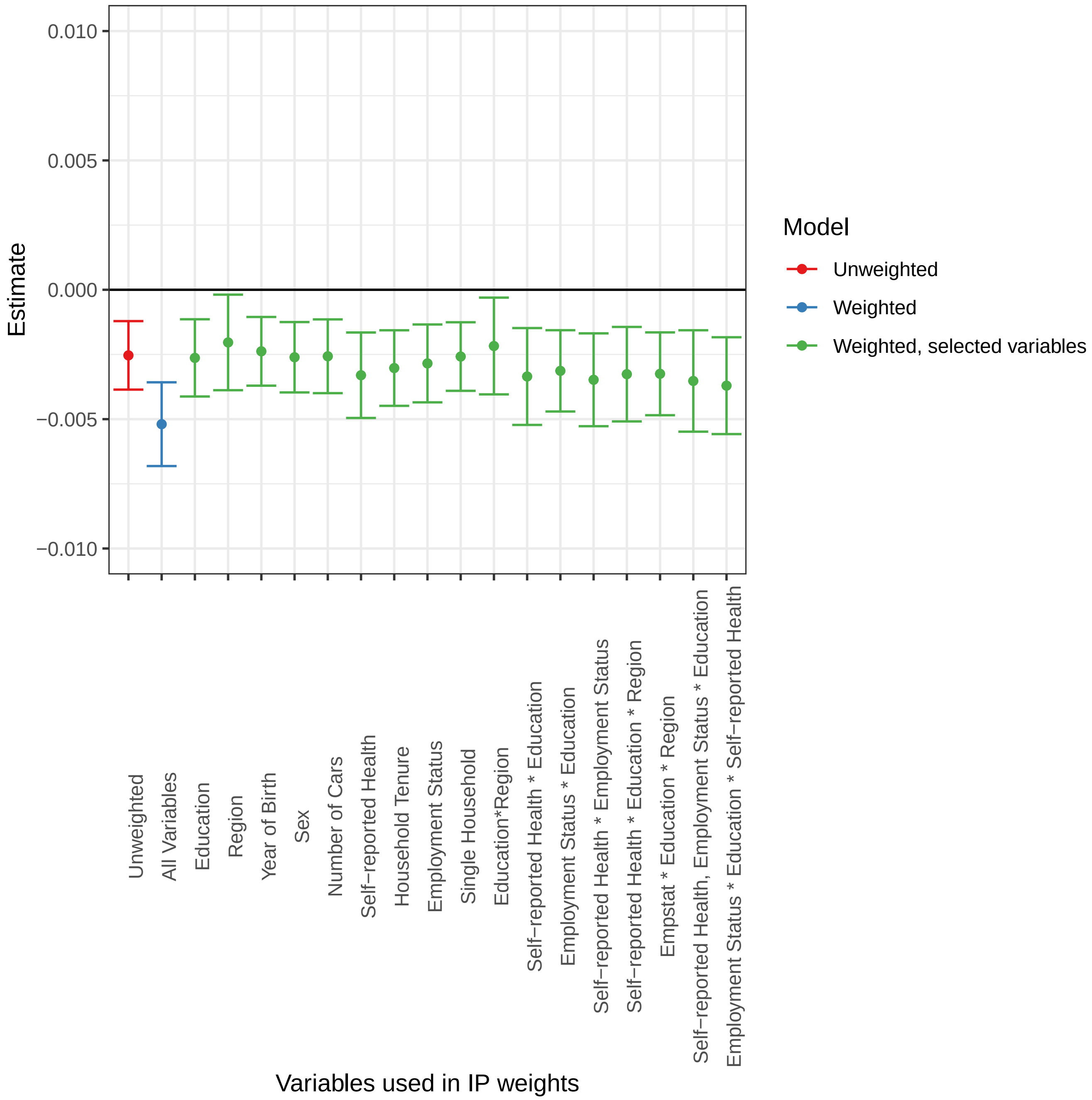

Figure S20: Association between *rs9861858* and type 1 diabetes, unweighted, weighted using our original IP weights ("All Variables"), and weighted using weights that were estimated on a sparser set of variables (as denoted by "labels")."*" refers to a model where the two variables, and all their respective two-way interactions, are included in estimating the weights.

Figure S21: **Simulated examples of how SNP associations become biased due to volunteering.** In the UKB, different phenotypes were simulated with different numbers of SNPs affecting the phenotype. Next, we simulated a selected sample from UKB data. The selected sample consists of the 5% that scored highest on S. The construction of S differs based on the scenario considered. Under Scenario 1, $S = \beta Y + \varepsilon$. Under Scenario 2a, $S = \beta Y + \beta PGI_Y + \varepsilon$. Under scenario 2b, $S = \beta Y - \beta PGI_Y + \varepsilon$. Each graph shows the association between the top SNP for the simulated phenotype as estimated within the selected sample. The black dotted line reflects the simulated true value of the association between the SNP and the phenotype. At higher levels of selection (higher values of β), the estimated association deviates further from the true association.

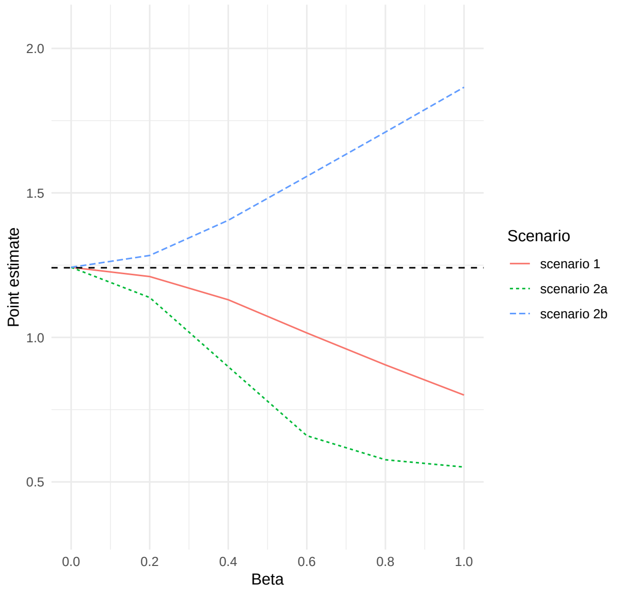

(a) 1 causal SNP

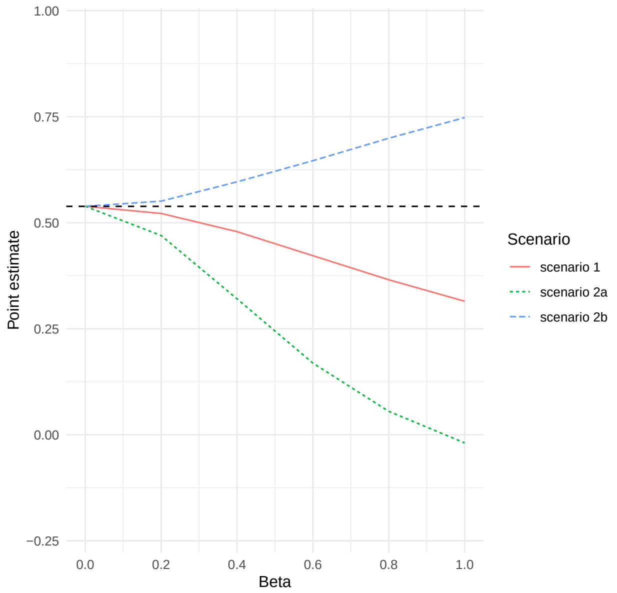

(b) 10 causal SNPs

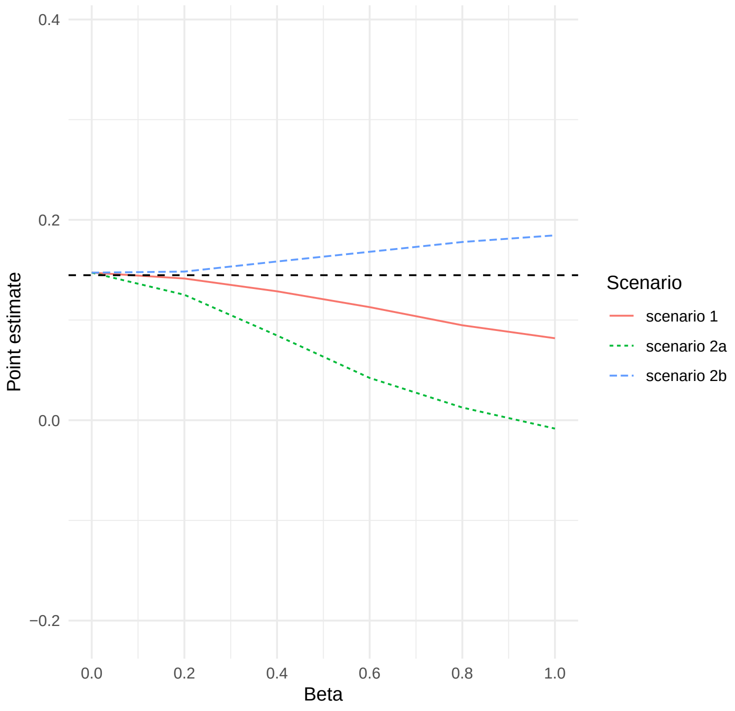

(c) 200 causal SNPs

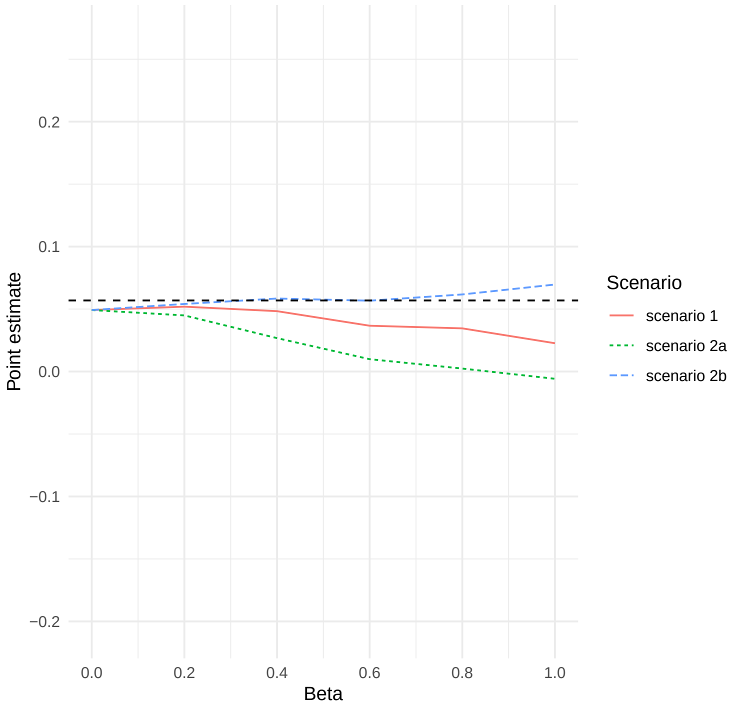

(d) 2000 causal SNPs

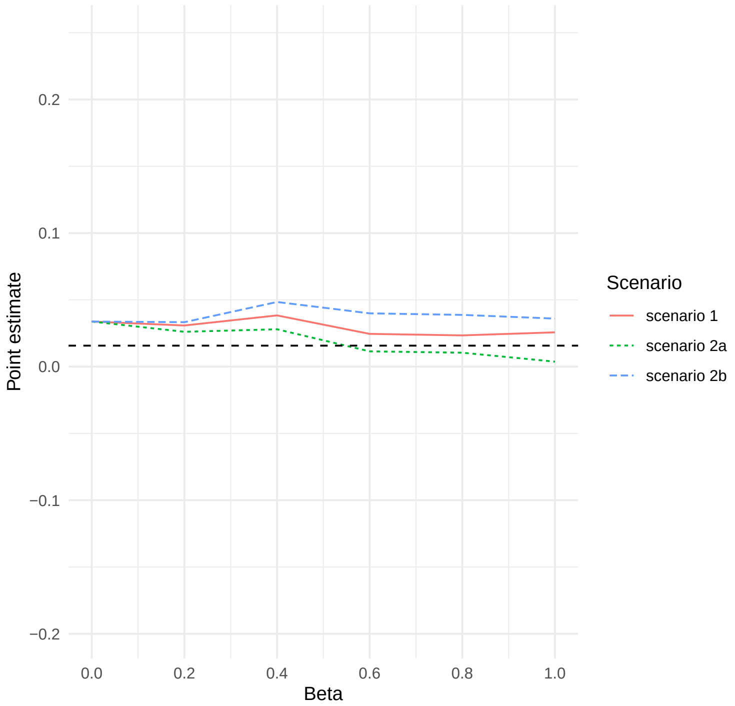

(e) All causal SNPs

Figure S22: **Simulated examples of how SNP-based heritabilities become biased due to volunteering.** In the UKB, different phenotypes were simulated with different numbers of SNPs affecting the phenotype, all with heritability of 0.2 (the black dotted line). Next, we simulated a selected sample from UKB data. The selected sample consists of the 5% that scored highest on S. The construction of S differs based on the scenario considered. Under Scenario 1, $S = \beta Y + \varepsilon$. Under Scenario 2a, $S = \beta Y + \beta PGI_Y + \varepsilon$. Under scenario 2b, $S = \beta Y - \beta PGI_Y + \varepsilon$. Within each sample, SNP-based heritability was estimated by running a GWAS on the simulated phenotype first, and next LD-score regression. At higher levels of selection (higher values of β), the estimated heritability deviates further from the true heritability.

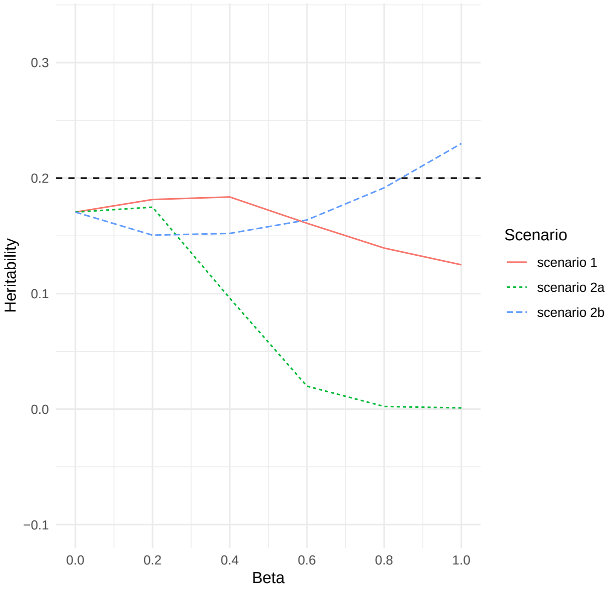

(a) 10 causal SNPs

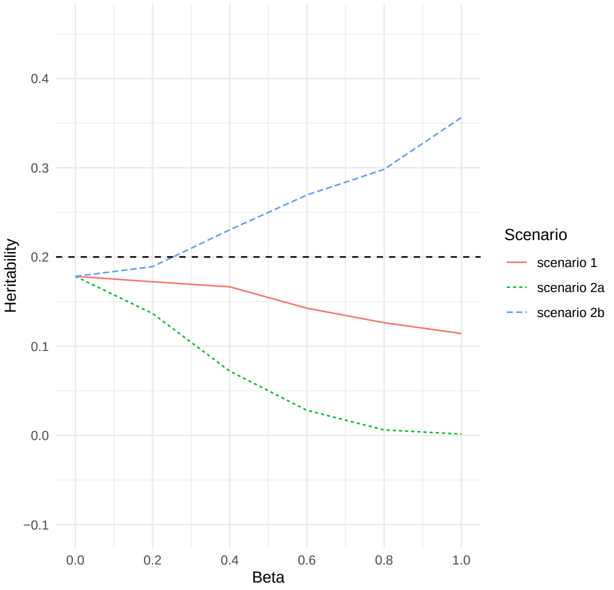

(b) 200 causal SNPs

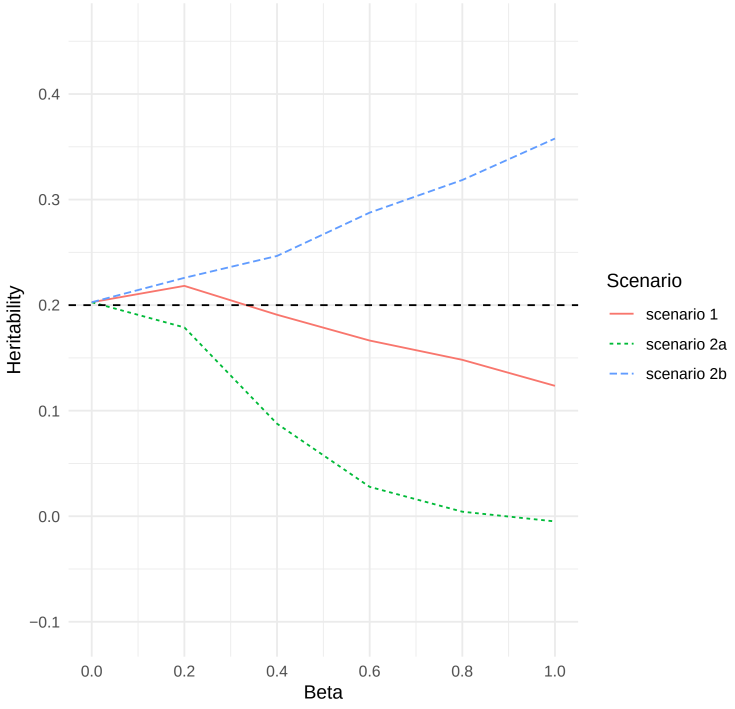

(c) 2000 causal SNPs

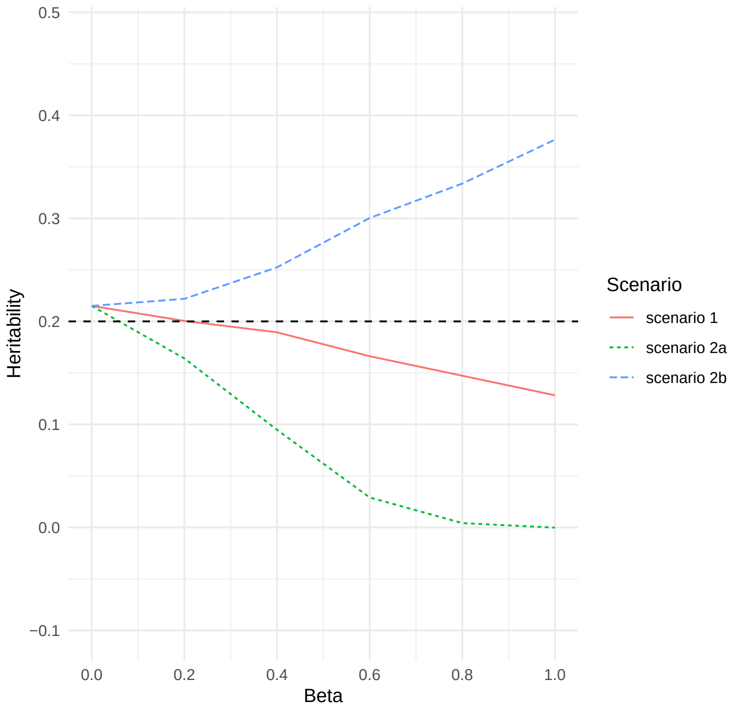

(d) All causal SNPs

Figure S23: **Simulated examples of how SNP associations become biased due to volunteering for binary phenotypes.** In the UKB, binary phenotypes were simulated with prevalence of 5% and 95% respectively. These phenotypes had either 1 causal SNP or 2000 causal SNPs. Next, we simulated a selected sample from UKB data. The selected sample consists of the 5% that scored highest on S. The construction of S differs based on the scenario considered. Under Scenario 1, $S = \beta Y + \varepsilon$. Under Scenario 2a, $S = \beta Y + \beta PGI_Y + \varepsilon$. Under scenario 2b, $S = \beta Y - \beta PGI_Y + \varepsilon$. Each graph shows the association between the top SNP for the simulated phenotype as estimated within the sample. The black dotted line reflects the value of the association in the representative population. At higher levels of selection (higher values of β), the estimated heritability deviates further from the true association.

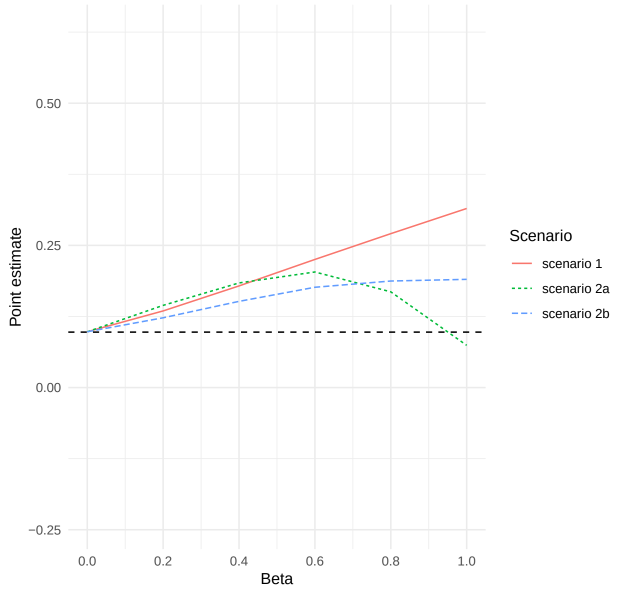

(a) 1 causal SNP, phenotype with 5% prevalence

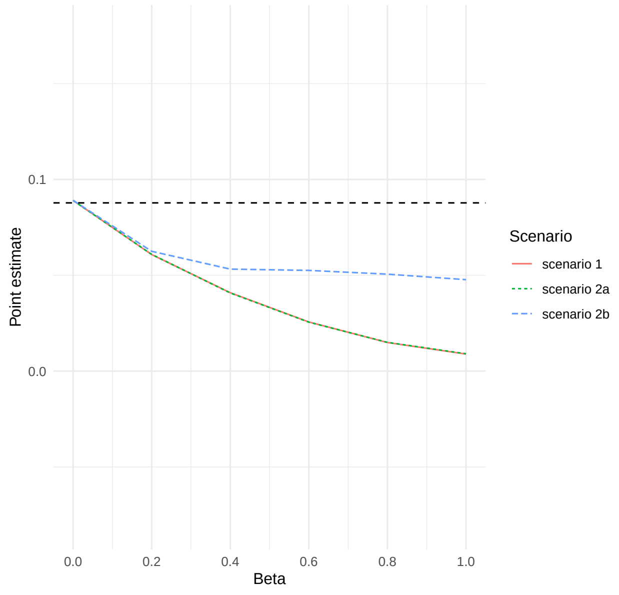

(b) 1 causal SNP, phenotype with 95% prevalence

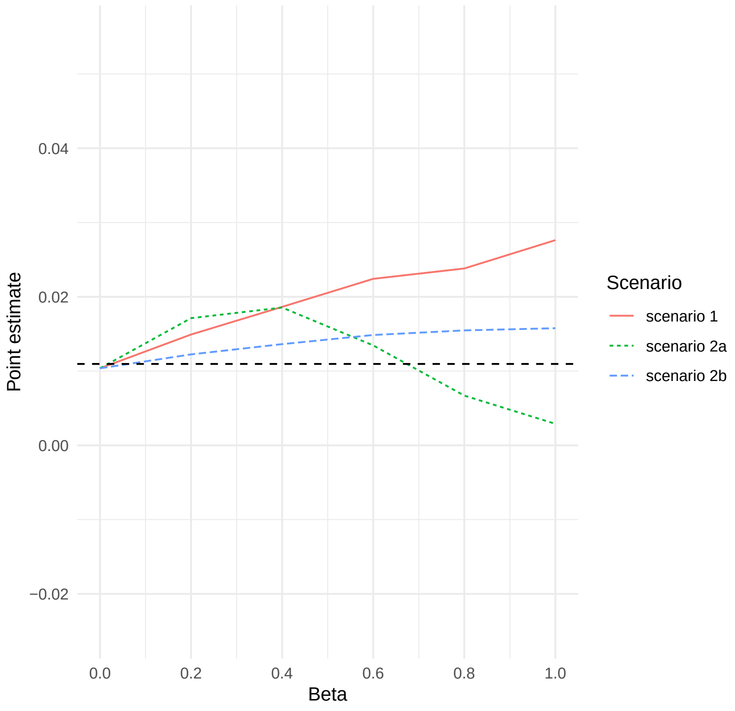

(c) 2000 causal SNPs, phenotype with 5% prevalence

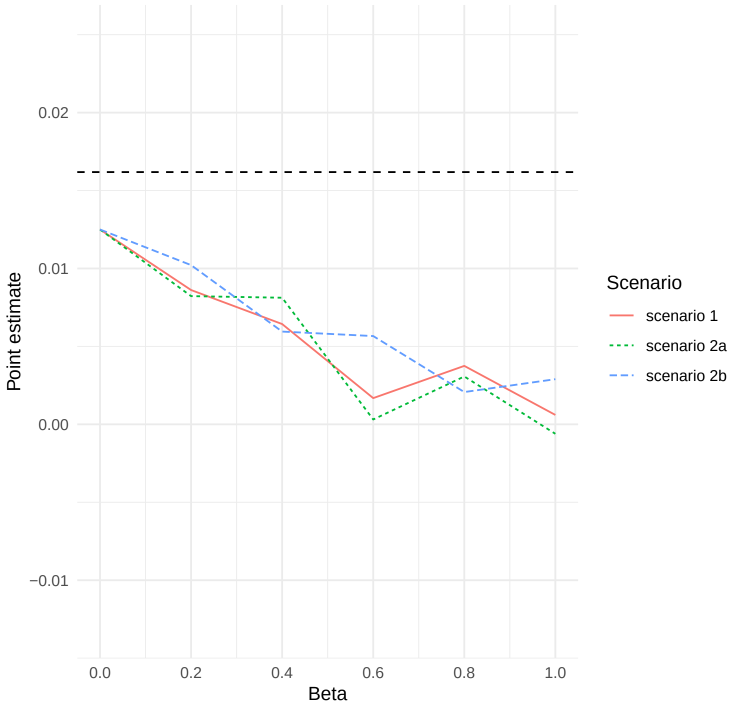

(d) 2000 causal SNPs, phenotype with 95% prevalence

Figure S24: **Simulated examples of how SNP-based heritabilities become biased due to volunteering for binary phenotypes.** In the UKB, binary phenotypes were simulated with prevalence of 5% and 95% respectively. Next, we simulated a selected sample from UKB data. The selected sample consists of the 5% that scored highest on S. The construction of S differs based on the scenario considered. Under Scenario 1, $S = \beta Y + \varepsilon$. Under Scenario 2a, $S = \beta Y + \beta PGI_Y + \varepsilon$. Under scenario 2b, $S = \beta Y - \beta PGI_Y + \varepsilon$. Within each sample, SNP-based heritability was estimated by running a GWAS on the simulated phenotype first, and next LD-score regression. At higher levels of selection (higher values of β), the estimated heritability deviates further from the heritability when no selection is present ($\beta = 0$)

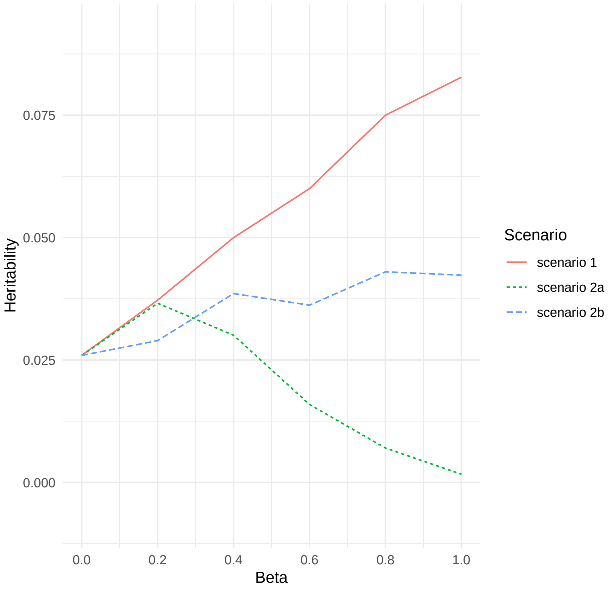

(a) 2000 causal SNP, phenotype with 5% prevalence

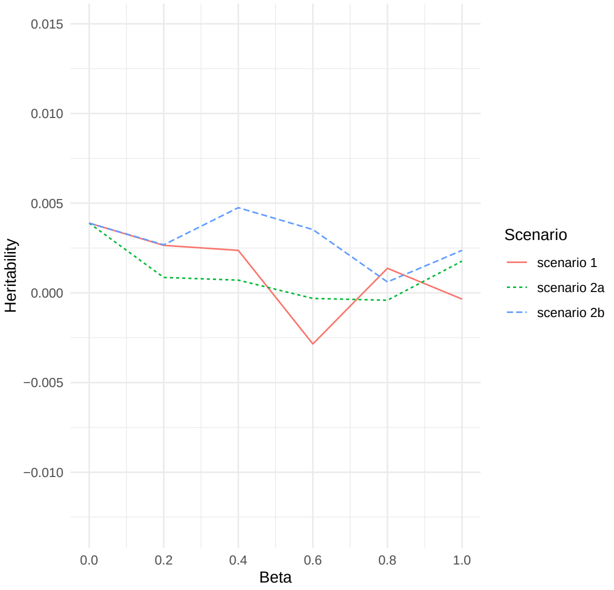

(b) 2000 causal SNP, phenotype with 95% prevalence

Supplementary References

[1] Speed D, Hemani G, Johnson MR, Balding DJ. Improved heritability estimation from genome-wide SNPs. The American Journal of Human Genetics. 2012;91(6):1011-21.

[2] van Alten S, Domingue BW, Faul J, Galama T, Marees AT. Reweighting UK Biobank corrects for pervasive selection bias due to volunteering. International Journal of Epidemiology. 2024;53(3):dyae054.

[3] Bulik-Sullivan BK, Loh PR, Finucane HK, Ripke S, Yang J, Patterson N, et al. LD Score regression distinguishes confounding from polygenicity in genome-wide association studies. Nature genetics. 2015;47(3):291-5.

[4] Klimentidis YC, Raichlen DA, Bea J, Garcia DO, Wineinger NE, Mandarino LJ, et al. Genome-wide association study of habitual physical activity in over 377,000 UK Biobank participants identifies multiple variants including CADM2 and APOE. International journal of obesity. 2018;42(6):1161-76.

[5] Okbay A, Wu Y, Wang N, Jayashankar H, Bennett M, Nehzati SM, et al. Polygenic prediction of educational attainment within and between families from genome-wide association analyses in 3 million individuals. Nature genetics. 2022;54(4):437-49.

[6] Buniello A, MacArthur JAL, Cerezo M, Harris LW, Hayhurst J, Malangone C, et al. The NHGRI-EBI GWAS Catalog of published genome-wide association studies, targeted arrays and summary statistics 2019. Nucleic acids research. 2019;47(D1):D1005-12.

[7] Sherry ST, Ward MH, Kholodov M, Baker J, Phan L, Smigielski EM, et al. dbSNP: the NCBI database of genetic variation. Nucleic acids research. 2001;29(1):308-11.

[8] Forgetta V, Manousaki D, Istomine R, Ross S, Tessier MC, Marchand L, et al. Rare genetic variants of large effect influence risk of type 1 diabetes. Diabetes. 2020;69(4):784-95.

[9] Michailidou K, Lindström S, Dennis J, Beesley J, Hui S, Kar S, et al. Association analysis identifies 65 new breast cancer risk loci. Nature. 2017;551(7678):92-4.
