# Supplementary material for: Correcting for volunteer bias in GWAS increases SNP effect sizes and heritability estimates
Source: Nat Commun. 2025 Apr 15;16:3578. doi: 10.1038/s41467-025-58684-8 (PMC12000612; doi:10.1038/s41467-025-58684-8)
Supplement: Supplementary file 2 — Description of Additional Supplementary Files [file 41467_2025_58684_MOESM2_ESM.pdf]

Description of Additional Supplementary Files

File Name: Supplementary Data 1
Description: Summary statistics for various phenotypes, unweighted (columns B an C) and weighted (columns D and E), with the relative change in the mean due to weighting expressed in column G)

File Name: Supplementary Data 2
Description: Suggestive hits for the GWAS on the IPWs. These SNPs tag approximately independent loci. Only SNPs that associate with P < 5*10^-5 are included.

File Name: Supplementary Data 3
Description: Sources of publicly available GWAS summary statistics used to estimate genetic correlations between the IP weights and various phenotypes.

File Name: Supplementary Data 4
Description: GWASs used to select top hits identified by studies that did not include the UKB

File Name: Supplementary Data 5
Description: Genes significantly associated with Type 1 Diabetes in WGWAS (P<5*10^-8), but not in GWAS, with effect sizes significantly different in WGWAS and GWAS (P<5*10^-8)

File Name: Supplementary Data 6
Description: SNPs significantly associated with Breast Cancer in WGWAS (P<5*10^-8), but not in GWAS, with effect sizes significantly different in WGWAS and GWAS (P<5*10^-8)

File Name: Supplementary Data 7
Description: Re-estimated effects of weighted and unweighted associations between 3 independent, newly identified top hits for Type 1 Diabetes, under various changes applied to the regression model

File Name: Supplementary Data 8
Description: Re-estimated effects of weighted and unweighted associations between 1 independent, newly identified top hit for Breast cancer, under various changes applied to the regression model
