# Supplementary material for: Correcting for volunteer bias in GWAS increases SNP effect sizes and heritability estimates
Source: Nat Commun. 2025 Apr 15;16:3578. doi: 10.1038/s41467-025-58684-8 (PMC12000612; doi:10.1038/s41467-025-58684-8)
Supplement: Supplementary file 4 — Reporting Summary [file 41467_2025_58684_MOESM4_ESM.pdf]

nature portfolio

Corresponding author(s): Sjoerd van Alten

Last updated by author(s): 2025/01/09

Reporting Summary

Nature Portfolio wishes to improve the reproducibility of the work that we publish. This form provides structure for consistency and transparency in reporting. For further information on Nature Portfolio policies, see our Editorial Policies and the Editorial Policy Checklist.

Statistics

For all statistical analyses, confirm that the following items are present in the figure legend, table legend, main text, or Methods section.

n/a	Confirmed	
☐	☒	The exact sample size (*n*) for each experimental group/condition, given as a discrete number and unit of measurement
☐	☒	A statement on whether measurements were taken from distinct samples or whether the same sample was measured repeatedly
☐	☒	The statistical test(s) used AND whether they are one- or two-sided<br>*Only common tests should be described solely by name; describe more complex techniques in the Methods section.*
☐	☒	A description of all covariates tested
☐	☒	A description of any assumptions or corrections, such as tests of normality and adjustment for multiple comparisons
☐	☒	A full description of the statistical parameters including central tendency (e.g. means) or other basic estimates (e.g. regression coefficient) AND variation (e.g. standard deviation) or associated estimates of uncertainty (e.g. confidence intervals)
☐	☒	For null hypothesis testing, the test statistic (e.g. *F*, *t*, *r*) with confidence intervals, effect sizes, degrees of freedom and *P* value noted<br>*Give P values as exact values whenever suitable.*
☒	☐	For Bayesian analysis, information on the choice of priors and Markov chain Monte Carlo settings
☒	☐	For hierarchical and complex designs, identification of the appropriate level for tests and full reporting of outcomes
☒	☐	Estimates of effect sizes (e.g. Cohen's *d*, Pearson's *r*), indicating how they were calculated

Our web collection on statistics for biologists contains articles on many of the points above.

Software and code

Policy information about availability of computer code

Data collection	No software was used for data collection
Data analysis	All code used for generating the results is available at \url{https://github.com/sjoerdvanalten/UKB_WGWAS}<br><br>We used the following software packages in data analysis:<br>R version 4.0.3 with packages ggplot2, tidyverse and ggExtra; R 4.1.0 with packages qqman, dplyr, plyr, ggplot2, ggrepel, stats, readxl, data.table, fastman; stringr, flexiblas, svMisc, estimatr, testit, doParallel, foreach, bigstatsr, BEDMatrix, lmtest, and AER;<br>Plink 1.9 (https://www.cog-genomics.org/plink/1.9);<br>QCTool 2.2 (https://www.well.ox.ac.uk/~gav/qctool_v2/);<br>ldsc 1.01 (https://github.com/bulik/ldsc);<br>BOLT-LMM_v2.4.1 (https://alkesgroup.broadinstitute.org/BOLT-LMM/BOLT-LMM_manual.html)<br>python 3.9.5 with packages pandas, numpy, re, os and subprocess.

For manuscripts utilizing custom algorithms or software that are central to the research but not yet described in published literature, software must be made available to editors and reviewers. We strongly encourage code deposition in a community repository (e.g. GitHub). See the Nature Portfolio guidelines for submitting code & software for further information.

Data

Policy information about availability of data

All manuscripts must include a data availability statement. This statement should provide the following information, where applicable:

- Accession codes, unique identifiers, or web links for publicly available datasets
- A description of any restrictions on data availability
- For clinical datasets or third party data, please ensure that the statement adheres to our policy

UK Biobank data is accessible upon request and approval by the UK Biobank committee (https://www.ukbiobank.ac.uk/). The IP weights developed here are available in the returned results catalogue under appliciation number 55154. Summary statistics of all GWAS and WGWAS analyses mentioned in this paper are available at https://github.com/sjoerdvanalten/UKB_WGWAS/tree/main/GWAS_Final and https://github.com/sjoerdvanalten/UKB_WGWAS/tree/main/WGWAS_Final, respectively.

Research involving human participants, their data, or biological material

Policy information about studies with human participants or human data. See also policy information about sex, gender (identity/presentation), and sexual orientation and race, ethnicity and racism.

Reporting on sex and gender	Most analyses were pooled across sex and gender, as our goal was to assess the effects of selection bias on such pooled GWAS summary statistics. Analyses for breast cancer and age at first birth were done in those of female genetic sex only. Data for age at first birth in males was not collected, and the prevalence of breast cancer in males was extremely low.
Reporting on race, ethnicity, or other socially relevant groupings	We restricted to those that were of White British ethnicity. Ethnicity groupings were self-reported at UK Biobank baseline (see UK Biobank data field 21000).
Population characteristics	UK Citizens of White British genetic ancestry aged 40-69 at UKB baseline (2006-2010).
Recruitment	Data was collected between 2006 and 2010 by the UK Biobank. They identified potential participants through the registry of the National Health Service, which covers virtually the whole UK population. Individuals living in proximity to an assessment centre and aged 40 to 69 at the start of the assessment period (which varies per assessment centre) received an invitation to participate by post. This UKB-eligible population consists of 9,234,453 individuals who received an invite, such that the overall acceptance rate was 5.45%. This low participation rate may lead to volunteer bias in GWAS results, which is our main bias of investigation in this paper.
Ethics oversight	The UK Biobank study was approved by the National Health Service's National Research Ethics Service. The present analysis was approved by the institutional review board of Vrije Universiteit Amsterdam (Amsterdam, the Netherlands).

Note that full information on the approval of the study protocol must also be provided in the manuscript.

Field-specific reporting

Please select the one below that is the best fit for your research. If you are not sure, read the appropriate sections before making your selection.

☐ Life sciences ☒ Behavioural & social sciences ☐ Ecological, evolutionary & environmental sciences

For a reference copy of the document with all sections, see nature.com/documents/nr-reporting-summary-flat.pdf

Behavioural & social sciences study design

All studies must disclose on these points even when the disclosure is negative.

Study description	This is a genome-wide association study (GWAS) of ten phenotypes conducted in the UK Biobank, that used weights to ensure population representativeness. All data used are quantitative.
Research sample	We used the UK Biobank (UKB), as it is one of the largest and most-used cohorts in GWAS analysis. The UK Biobank is a large-scale biomedical database containing genetic, phenotypic, and health-related data from approximately 502,485 participants aged 40-69 years, recruited across the United Kingdom between 2006 and 2010. Because the sample relied on volunteers, it is not representative of its underlying sampling population. We took all UKB respondents at baseline. We dropped individuals for whom genetic data was not collected, and restricted the UKB to individuals who identified as "white British" and were of genetic European ancestry. (dropping 92,887). We also dropped respondents that did not meet the standard requirements regarding genetic data quality control (dropping 26,406). Last, we dropped 6,292 respondents (1.6%) for whom inverse probability weights were unavailable, typically because of missing variables.
Sampling strategy	We defined our sample in a similar way to other published GWAS analyses of the UK Biobank.
Data collection	We used secondary data collected by the UK Biobank.

Timing	The UK Biobank sampled its respondents between 2006 and 2010.
Data exclusions	See above and supplementary figure 1
Non-participation	The UK Biobank had a response rate of 5.45%
Randomization	Participants were not allocated into experimental groups

Reporting for specific materials, systems and methods

We require information from authors about some types of materials, experimental systems and methods used in many studies. Here, indicate whether each material, system or method listed is relevant to your study. If you are not sure if a list item applies to your research, read the appropriate section before selecting a response.

Materials & experimental systems

n/a	Involved in the study
☒	☐ Antibodies
☒	☐ Eukaryotic cell lines
☒	☐ Palaeontology and archaeology
☒	☐ Animals and other organisms
☒	☐ Clinical data
☒	☐ Dual use research of concern
☒	☐ Plants

Methods

n/a	Involved in the study
☒	☐ ChIP-seq
☒	☐ Flow cytometry
☒	☐ MRI-based neuroimaging

Plants

Seed stocks	*Report on the source of all seed stocks or other plant material used. If applicable, state the seed stock centre and catalogue number. If plant specimens were collected from the field, describe the collection location, date and sampling procedures.*
Novel plant genotypes	*Describe the methods by which all novel plant genotypes were produced. This includes those generated by transgenic approaches, gene editing, chemical/radiation-based mutagenesis and hybridization. For transgenic lines, describe the transformation method, the number of independent lines analyzed and the generation upon which experiments were performed. For gene-edited lines, describe the editor used, the endogenous sequence targeted for editing, the targeting guide RNA sequence (if applicable) and how the editor was applied.*
Authentication	*Describe any authentication procedures for each seed stock used or novel genotype generated. Describe any experiments used to assess the effect of a mutation and, where applicable, how potential secondary effects (e.g. second site T-DNA insertions, mosiacism, off-target gene editing) were examined.*
